# Stereoselective Biginelli-like reaction catalyzed by a chiral phosphoric acid bearing two hydroxy groups

**DOI:** 10.3762/bjoc.16.155

**Published:** 2020-07-31

**Authors:** Xiaoyun Hu, Jianxin Guo, Cui Wang, Rui Zhang, Victor Borovkov

**Affiliations:** 1 College of Chemistry and Materials South-Central University for Nationalities, Wuhan, China; 2 Department of Chemistry and Biotechnology, Tallinn University of Technology, Akadeemia tee 15, Tallinn 12618, Estonia

**Keywords:** asymmetric Biginelli-like reaction, chiral 1,1,4,4-tetraphenylbutanetetraol, chiral phosphoric acid

## Abstract

To develop new efficient stereoselective catalysts for Biginelli-like reactions, a chiral phosphoric acid bearing two hydroxy groups derived from ʟ-tartaric acid was successfully synthesized via highly regioselective transformations of enantiopure 1,1,4,4-tetraphenylbutanetetraol. The obtained catalyst effectively catalyzed Biginelli-like reactions with moderate to good enantioselectivities. Control experiments indicated that the presence of the two hydroxy groups were indispensable for achieving a high enantioselectivity.

## Introduction

Dihydropyrimidinethiones (DHPMs) are an important class of heterocyclic compounds featuring in a large number of natural and artificial compounds possessing various biological activities, and serving as key intermediates for the synthesis of medical drugs [1–4]. In the last decade, chiral DHPMs showed valuable pharmacological properties such as antiviral, antitumor, antibacterial, anti-inflammatory, and antihypertensive effects [5–6]. However, the individual enantiomers of DHPMs were found to exhibit different or even opposite pharmaceutical activities [7–8]. For example, (*S*)-monastrol is 15-fold more effective in the inhibition of Eg 5 ATPase than its enantiomer, (*R*)-monastrol [9]. As more reports on the enantiospecific biological activity of DMPMs came to light, the development of an efficient and reliable asymmetric synthesis of enantiopure DHPMs becomes an urgent and paramount task.

In connection with this, multicomponent Biginelli and Biginelli-like reactions of aldehydes, urea/thiourea, and enolizable carbonyls revealed as very efficient tools to prepare DHPMs [3]. Although the Biginelli reaction was firstly discovered over a century ago [10], little attention was given to asymmetric pathways. Only in 2003, Juaristi and Muñoz-Muñiz have reported the reaction of benzaldehyde, urea, and methyl acetoacetate using a chiral amide with CeCl_3_ for the first time. However, only 40% enantiomeric excess (ee) was obtained [11]. Then, Zhu described the efficient asymmetric Biginelli reaction with a chiral ytterbium catalyst to provide excellent enantioselectivities up to >99% ee [12]. Later, Gong reported the first organocatalytic Biginelli reaction using 1,1'-bi-2-naphthol (BINOL) derived chiral phosphoric acids as catalysts, furnishing the DHPMs with up to 97% ee [13–14].

Though the BINOL-derived chiral phosphoric acids were successfully applied in the Biginelli reactions, the costs are essentially high because of complex preparative procedures, especially for the introduction of a substituent at the 3,3'-position of BINOL [15]. Besides BINOL, 2,2-dimethyltetraphenyl-1,3-dioxolane-4,5-dimethanol (TADDOL) was also widely used as a *C*_2_ chiral diol. However, TADDOL-derived phosphoric acids were not comprehensively investigated as catalysts in Biginelli and Biginelli-like reactions yet. The first example of their successful application was reported in 2005 by the Akiyama’s group [16]. This work clearly demonstrated the potential of these catalysts in enantioselective transformations. An additional important point of this work was the use of enantiopure tartaric acid as a precursor for the catalyst preparation being a conventional natural compound making its application highly attractive from the viewpoint of sustainability and green chemistry. Recently, our group reported an asymmetric Biginelli reaction catalyzed by a new chiral phosphoric acid derived from natural tartaric acid, that yielded a high enantioselectivity (up to 99% ee) [17]. This new chiral phosphoric acid featured a facile preparation and low cost. In order to further develop this new chiral phosphoric acid as an asymmetric catalyst, herein its catalytic efficacy in the Biginelli-like reactions of aldehydes, benzylthiourea, and cyclohexanone was explored, and moderate to good enantioselectivities (up to 95% ee) were obtained. Furthermore, a control experiment was designed to disclose the indispensability of the two hydroxy groups for achieving high enantioselectivities of the Biginelli-like reaction.

## Results and Discussion

**Scheme 1:**
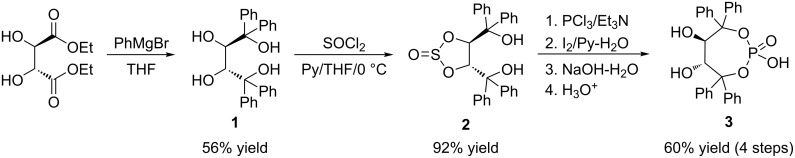
Synthesis of chiral phosphoric acid **3**.

Enantiomerically pure 1,1,4,4-tetrasubstituted butanetetraol **1** is a parent compound of TADDOLs, which conventionally are obtained from enantiopure tartaric acids. The hydroxy group composition of **1** ensures its diverse reactivity. In 2010, our group established a convenient access [18] to **1** via a direct alkylation of diethyl tartrates by using PhMgBr followed by further modifications involving highly regioselective 1,4-cycloetherization [19], 2,3-spiroboration [20], 2,3-sulfitation [21], and 2,3-methylation [22] of **1**. Therefore, the strategy for the catalyst’s preparation was based on the established regioselective 2,3-sulfitation reaction of **1** and ready hydrolysis of the corresponding sulfite ester **2** in NaOH solution to yield the chiral phosphoric acid **3** bearing two free hydroxy groups at the 2 and 3- positions, respectively, as shown in Scheme 1.

Our previous work indicated that the phosphoric acid derivative **3** was able to catalyze the asymmetric Biginelli reaction of unsubstituted and electron-rich aromatic aldehydes [17]. To further expand the application of this type of chiral phosphoric acids, the asymmetric Biginelli-like catalytic reaction was examined. Firstly, coupling of benzaldehyde (**4a**), cyclohexanone, and *N*-benzylthiourea was set as a model reaction (Table 1). Screening of solvents indicated that chiral phosphoric acid **3** stereoselectively promoted this reaction in aprotic solvents (Table 1, entries 1–6), while in protic solvent, such as ethanol, the racemic product was obtained (Table 1, entry 7). To our pleasure, the solvent screening showed that the target product could be obtained in 95% ee catalyzed by **3** in CHCl_3_ (Table 1, entry 4). However, acidic and basic additives [23–24] resulted in a decrease of the enantioselectivity (Table 1, entries 10–12), or even to racemization in the case of TFA (Table 1, entry 13). The role of the reaction temperature was also investigated (Table 1, entries 8 and 9) and it was found that decreasing or raising the temperature was unfavorable for both the yield and enantioselectivity. Based on these results, the optimal conditions were selected as follows: 10 mol % **3** as the catalyst, CHCl_3_ as the reaction solvent at 50 °C for 6 days.

**Table 1:** Optimization of reaction conditions^a^.

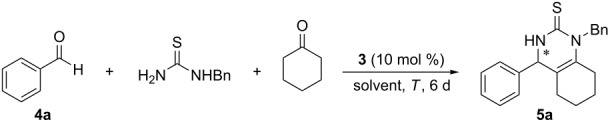					

entry	solvent	additive	*T* [°C]	yield [%]^b^	ee [%]^c^

1	toluene	–	50	76	89
2	THF	–	50	43	51
3	acetone	–	50	56	44
**4**	**CHCl_3_**	–	**50**	**85**	**95**
5	CH_2_Cl_2_	–	50	75	76
6	CH_3_CN	–	50	43	32
7	EtOH	–	50	82	0
8	CHCl_3_	–	30	60	33
9	CHCl_3_	–	70	50	47
10	CHCl_3_	Py	50	32	16
11	CHCl_3_	NaHCO_3_	50	30	12
12	CHCl_3_	PhCOOH	50	87	44
13	CHCl_3_	CF_3_COOH	50	91	0

^a^Reaction was carried out on a 0.2 mmol scale, the ratio of **4a/***N*-benzylthiourea**/**cyclohexanone was 1.5:1.0:5.0 and 10 mol % of **3**. ^b^Isolated yields. ^c^The ee was determined by HPLC.

With the optimal conditions in hand, the scope of aromatic aldehydes **4** was explored. As shown in Table 2, a range of aromatic aldehydes gave the target products in high yields with moderate to good enantioselectivities in general. However, it should be noted that the enantiomer self-disproportionation effect may take place during the purification by column chromatography especially in the case of products having strongly electronegative groups [25–29].

In particular, benzaldehyde (**4a**) and 3-bromobenzaldehyde (**4c**) gave products **5a** and **5c** with 95% and 91% ee, respectively (Table 2, entries 1 and 3). As further can be seen from Table 2, the position of substituents had a dramatic influence on the enantioselectivity (Table 2, entries 2–4). This may be attributable to the different inductive effects induced by the *o-*, *p-*, and *m-*bromo substituent in the aromatic aldehydes **4b**–**d** and steric hindrance. Both the electron-donating and electron-withdrawing groups in the 2-position were unfavorable for the enantioselectivity (Table 2, entries 2, 9, and 10). The presence of electron-donating groups in the 4-position was more favorable for the enantioselectivity (Table 2, entries 4–6, and 8) except for the sterically demanding *tert*-butyl group (Table 2, entry 12). Obviously, steric effects in the aromatic aldehydes played a key role for the enantioselectivity of the reaction.

**Table 2:** Results of the Biginelli-like reaction with various aromatic aldehydes catalyzed by **3a**^a^.

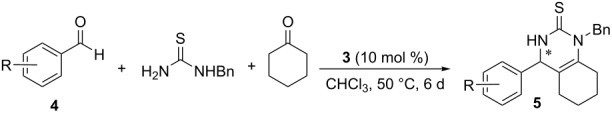				

entry	R	product	yield [%]^b^	ee [%]^c^

1	H	**5a**	85	95
2	2-Br	**5b**	86	77
3	3-Br	**5c**	83	91
4	4-Br	**5d**	90	87
5	4-Me	**5e**	88	80
6	4-OMe	**5f**	85	83
7	4-F	**5g**	89	73
8	4-NO_2_	**5h**	79	42
9	2-NO_2_	**5i**	76	23
10	2-Cl	**5j**	72	53
11	4-*t-*Bu	**5k**	73	20

^a^Reaction was carried out on a 0.2 mmol scale, the ratio of **4/***N*-benzylthiourea**/**cyclohexanone was 1.5:1.0:5.0 and 10 mol % of **3**. ^b^Isolated yields. ^c^The ee was determined by HPLC.

**Scheme 2:**
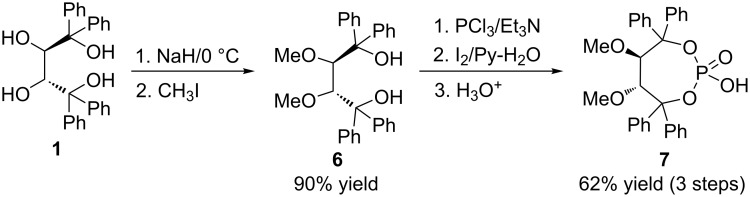
Synthesis of methylated chiral phosphoric acid **7**.

To further confirm the importance of two secondary free hydroxy groups in **3** for this reaction, the methylated derivative **7** was synthesized (Scheme 2). As shown in Scheme 2, first (2*R*,3*R*)-**1** was subjected to highly regioselective 2,3-dimethylation [23] with NaH/MeI to give product (2*R*,3*R*)-**6**. Then the corresponding dimethylated chiral phosphoric acid **7** was synthesized in a similar manner as described in [17].

**Scheme 3:**
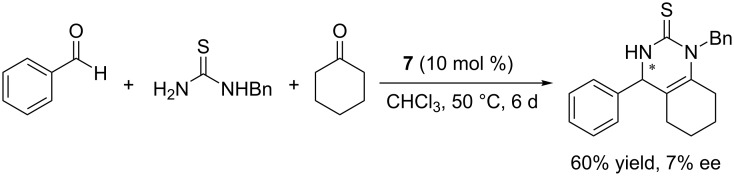
Control experiment with catalyst **7**.

Next, the model reaction of **4a**, cyclohexanone, and *N*-benzylthiourea catalyzed by **7** under the optimized conditions was examined and the corresponding product **5a** was obtained in 60% yield and 7% ee (Scheme 3). Compared to the dihydroxy compound **3**, both the yield and enantioselectivity of the reaction dropped dramatically, hence confirming our initial proposal that the free hydroxy groups in the chiral phosphoric acid **3** played an important role in this enantioselective transformation.

**Figure 1:**
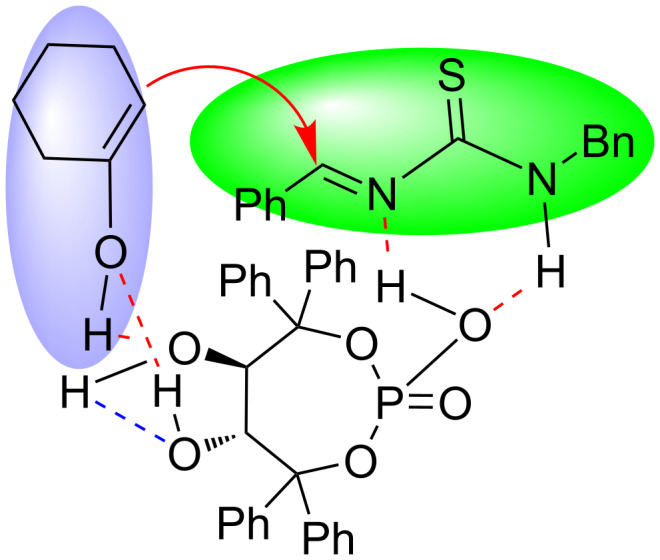
A plausible chiral transition-state structure in the Biginelli-like reaction catalyzed by phosphoric acid **3**.

Based on these results, a plausible transition-state structure was proposed. As shown in Figure 1, the chiral phosphoric acid **3** activated the imine derivative, which in turn was formed by the condensation of benzaldehyde with *N*-benzylthiourea. The two hydroxy groups formed five-numbered intramolecular (blue dotted line) and seven-numbered intermolecular (red dotted lines) H bondings with the enolizable ketone, respectively. This rigid chiral transition-state structure favored the stereoselective attack of the enol on the imine. Once the two hydroxy groups were etherified, the loss of the rigid structure would lead to low enantioselectivity. Additionally, this explains that more polar or protic solvents and strong acidic additives also destroyed the H-bondings resulting in decreased enantioselectivities or even racemization (Table 1, entries 7 and 13).

## Conclusion

In summary, a new phosphoric acid bearing two free hydroxy groups was synthesized based on a highly regioselective transformation of chiral 1,1,4,4-tetraphenylbutanetetraol obtained from natural tartaric acid. The chiral phosphoric acid was successfully applied as asymmetric catalyst in the Biginelli-like reaction affording the products in good yields and enantioselectivities of up to 95%. A control experiment indicated that the two free hydroxy groups in phosphoric acid **3** played a pivotal role in improving the stereoselectivity. A plausible activation model and reaction pathways of the stereogenic step in the phosphoric acid **3**-catalyzed Biginelli-like reaction was proposed. This promising result prompted us to expand the applicability of this kind of catalysts to other types of asymmetric reactions, which is underway in our laboratory.

## Experimental

**Materials and general methods: **^1^H and ^13^C NMR spectra were performed on a Varian Mercury VS 300 or Bruker Avance III 400. Optical rotations were measured on a PE-341 Mc polarimeter. Melting points were determined on a VEB Wägetechnik Rapio PHMK05 instrument, and are uncorrected. Enantiomeric excess (ee) values were analyzed by a Thermo UltiMate 3000 HPLC and SHIMADZU LC-20 AR at room temperature with *n*-hexane/isopropanol as eluent. Diethyl ʟ-tartrate was prepared from ʟ-tartaric acid and ethanol. THF was freshly distilled after refluxing with Na, while SOCl_2_, pyridine, PCl_3_, and I_2_ were purchased and used directly. Commercially available starting materials were used without further purification if not specified otherwise. Chiral phosphoric acid **3** was prepared according to a previous method [18].

**Preparation of 1** [18]**:** A THF solution of ʟ-diethyl tartrate (6.2 g, 30 mmol) was slowly added dropwise into a freshly prepared PhMgBr solution. After the addition, the mixture was heated to reflux for 2 h. Then, 100 mL of saturated aqueous NH_4_Cl were added into the mixture after cooling to rt. The resulting solution was extracted with EA (20 mL × 3), dried, and concentrated. The residue was recrystallized from 80% ethanol (ethanol/H_2_O 4:1, v/v) to give 7.2 g of (2*R*,3*R*)-**1**. Yield 56%; mp 149–151 °C; [α]_D_^25^ +153.9 (*c* 0.5, CHCl_3_); ^1^H NMR (CDCl_3_, 400 MHz) δ 7.31–7.16 (m, 20H, Ar-H), 4.66 (d, *J* = 7.3 Hz, 2H, OH), 4.42 (d, *J* = 4.8 Hz, 2H, CH), 3.78 (d, *J* = 5.4 Hz, 2H, OH); ^13^C NMR (CDCl_3_, 75 MHz) δ 144.1, 143.9, 128.7, 128.5, 127.4, 127.3, 126.1, 125.0, 81.8, 71.2.

**Preparation of 6** [22]**:** A dried round-bottomed flask was charged with (2*R*,3*R*)-**1** (0.916 g, 2.0 mmol), sodium hydride (0.105 g, 4.2 mmol), and dried THF (16 mL). The mixture was stirred at rt for 2 h, and then methyl iodide (0.61 g, 4.3 mmol) was added and the mixture stirred at rt for 6 h. Then, distilled water (12 mL) and diethyl ether (15 mL) were added, the organic phase was separated, dried, and concentrated. The residue was recrystallized from ethanol to afford 0.776 g (2*R*,3*R*)-**6**. Yield 90%; mp 124–125 °C; [α]_D_^25^ −151 (*c* 0.8, CHCl_3_); ^1^H NMR (300 MHz, CDCl_3_) δ 7.63–7.55 (m, 8H, Ph-H), 7.43 (t, *J* = 7.5 Hz, 4H, Ph-H), 7.32–7.12 (m, 8H, Ph-H), 4.91 (s, 2H, O-H), 4.43 (s, 2H, C-H), 2.53 (s, 6H, 2CH_3_); ^13^C NMR (75 MHz, CDCl_3_) δ 145.9, 145.0, 128.7, 128.2, 127.5, 127.0, 126.3, 126.2, 126.1, 85.62, 85.57, 80.19, 61.35, 61.26.

**Preparation of 7:** In a similar manner as described in [17], under Ar, a dried three-necked round-bottomed 250 mL flask equipped with a magnetic stirring bar, reflux condenser with oil seal, and a 100 mL pressure-equalizing dropping funnel was charged with 10 mL of dry THF and 3.2 mL of NEt_3_ (22 mmol). The flask was placed in an ice-bath and PCl_3_ (1 mL) added, and the resulting mixture was stirred at 0 °C for 20 min. Then, the dropping funnel was charged with a THF solution of (2*R*,3*R*)-**6** (6.1 g, 13 mmol) and added dropwise to the mixture. After the addition, the mixture was stirred at 0 °C for 1 h, warmed to rt, and stirred for an additional 0.5 h. Then, 1.5 mL of H_2_O were added, followed by 10.9 g of I_2_, and 7.1 mL of pyridine and the mixture stirred for another 1 h. Afterwards, the mixture was poured into a saturated NaHSO_3_ solution and stirred to remove the excess I_2_. Finally, 2 M HCl was added to adjust the pH to 2–3. The solution was extracted with Et_2_O (20 mL × 3), dried and concentrated. Recrystallization from ethanol furnished 4.2 g of (5*R*,6*R*)-2-hydroxy-5,6-dimethoxy-4,4,7,7-tetraphenyl-1,3,2-dioxaphosphepane 2-oxide (**7**). Yield 62%; mp 188–190 °C; ^1^H NMR (400 MHz, DMSO-*d*_6_) δ 7.57 (d, *J* = 7.5 Hz, 4H, Ar-H), 7.34 (t, *J* = 7.4 Hz, 4H, Ar-H), 7.23 (d, *J* = 6.7 Hz, 6H, Ar-H), 7.16–7.03 (m, 6H, Ar-H), 4.53 (s, 2H, CH), 3.41 (d, *J* = 27.2 Hz, 6H, CH_3_); ^13^C NMR (101 MHz, DMSO-*d*_6_) δ 146.7, 144.3, 128.5, 127.6, 127.5, 127.3, 126.7, 126.3, 87.7, 86.3, 58.7; Anal. calcd for C_30_H_29_O_6_P: C, 69.76; H, 5.66; found: C, 69.67; H, 5.59.

**Typical procedure for asymmetric catalyzed Biginelli-like reactions:** In a similar manner as described in [17], after a solution of an aromatic aldehyde **4** (0.3 mmol, 1.5 equiv), *N*-benzylthiourea (0.2 mmol), and chiral phosphoric acid **3** (0.02 mmol) in CHCl_3_ (1.5 mL) was stirred at 25 °C for 2 h, cyclohexanone (1 mmol) was added. The resulting mixture was warmed to 50 °C, stirred for 6 days, and then silica gel was added. After removal of the solvent, the residue was purified by column chromatography using petroleum ether/ethyl acetate 6:1–3:1.

## Supporting Information

File 1 Experimental data and copies of ^1^H NMR and ^13^C NMR spectra.
