# Supplementary material for: Stereoselective Biginelli-like reaction catalyzed by a chiral phosphoric acid bearing two hydroxy groups
Source: Beilstein J Org Chem. 2020 Jul 31;16:1875–80. doi: 10.3762/bjoc.16.155 (PMC7404148; doi:10.3762/bjoc.16.155)
Supplement: File 1 — Experimental data and copies of 1H NMR and 13C NMR spectra. [file Beilstein_J_Org_Chem-16-1875-s001.pdf]

BEILSTEIN JOURNAL OF ORGANIC CHEMISTRY

Supporting Information

for

Stereoselective Biginelli-like reaction catalyzed by a chiral phosphoric acid bearing two hydroxy groups

Xiaoyun Hu, Jianxin Guo, Cui Wang, Rui Zhang and Victor Borovkov

Beilstein J. Org. Chem. **2020,** *16,* 1875–1880. doi:10.3762/bjoc.16.155

Experimental data and copies of ^{1}H NMR and ^{13}C NMR spectra

License and Terms: This is a supporting information file under the terms of the Creative Commons Attribution License (http://creativecommons.org/licenses/by/4.0). Please note that the reuse, redistribution and reproduction in particular requires that the authors and source are credited.
The license is subject to the *Beilstein Journal of Organic Chemistry* terms and conditions: (https://www.beilstein-journals.org/bjoc)

Table of contents

I. Experimental data S1

II. NMR spectra...S7

III. HPLC analysis S23

I Experimental data

(*R*)-1-Benzyl-4-phenyl-3,4,5,6,7,8-hexahydroquinazoline-2(1*H*)-thione (5a)

Yield: 85%, m.p.: 183-185 ℃, ^{1}H NMR (400 MHz, Chloroform-*d*) δ 7.34 (t, *J* = 6.4 Hz, 5H), 7.28 (t, *J* = 7.8 Hz, 5H), 7.03 (s, 1H), 5.87 (d, *J* = 15.5 Hz, 1H), 5.31 (d, *J* = 9.3 Hz, 1H), 4.76 (s, 1H), 2.30–2.17 (m, 2H), 1.89–1.72 (m, 2H), 1.71–1.46 (m, 4H). ^{13}C NMR (101 MHz, Chloroform-*d*) δ 177.4, 141.4, 138.1, 130.1, 128.9, 128.6, 128.3, 127.2, 127.1, 126.4, 113.5, 59.3, 51.0, 26.7, 25.6, 22.8, 21.2. Enantiomeric excess: 95%, determined by HPLC (Daicel Chirapak AD-H, hexane/*i*-PrOH = 70/30, flow rate 1.0 mL/min): T_R = 9.617 min (minor), T_R = 18.415 min (major).

(*R*)-1-Benzyl-4-(2-bromophenyl)-3,4,5,6,7,8-hexahydroquinazoline-2(1*H*)-thione (5b)

Yield: 86%, m.p.: 179-180 ℃, ^{1}H NMR (400 MHz, Chloroform-*d*) δ 7.57 (dd, *J* = 7.8, 1.1 Hz, 1H), 7.34–7.26 (m, 4H), 7.25–7.22 (m, 2H), 7.20–7.13 (m, 2H), 7.05 (s, 1H), 6.05 (d, *J* = 14.2 Hz, 1H), 5.18 (s, 1H), 5.08 (d, *J* = 16.1 Hz, 1H),

2.28 (t, J = 5.7 Hz, 2H), 1.98–1.74 (m, 2H), 1.70–1.54 (m, 3H), 1.47–1.37 (m, 1H). ^{13}C NMR (101 MHz, Chloroform-*d*) δ 177.7, 139.0, 137.9, 133.3, 131.9, 130.0, 129.2, 128.6, 128.3, 127.2, 126.7, 123.8, 112.6, 57.4, 51.0, 26.6, 25.7, 22.8, 21.2. Enantiomeric excess: 77%, determined by HPLC (Daicel Chirapak AD-H, hexane/*i*-PrOH = 70/30, flow rate 1.0 mL/min): T_R = 9.265 min (minor), T_R = 15.480 min (major).

(*R*)-1-Benzyl-4-(3-bromophenyl)-3,4,5,6,7,8-hexahydroquinazoline-2(1*H*)-thione (5c)

Yield: 83%, m.p.: 108-110 ℃, ^{1}H NMR (400 MHz, Chloroform-*d*) δ 7.52 – 7.37 (m, 5H), 7.31 (t, J = 6.4 Hz, 3H), 7.23 (d, J = 6.4 Hz, 2H), 6.00 (d, J = 7.6 Hz, 1H), 5.22 (d, J = 8.3 Hz, 1H), 4.74 (s, 1H), 2.26 (s, 2H), 1.94–1.73 (m, 2H), 1.71–1.38 (m, 4H). ^{13}C NMR (151 MHz, Chloroform-*d*) δ 176.0, 142.7, 136.8, 130.4, 129.5, 129.0, 127.7, 126.1, 125.3, 124.9, 122.0, 111.8, 57.6, 49.8, 25.6, 24.5, 21.6, 20.1. Enantiomeric excess: 91%, determined by HPLC (Daicel Chirapak AD-H, hexane/*i*-PrOH = 70/ 30, flow rate 1.5 mL/min): T_R = 6.823 min (minor), T_R = 16.628 min (major).

(*R*)-1-Benzyl-4-(4-bromophenyl)-3,4,5,6,7,8-hexahydroquinazoline-2(1*H*)-thione (5d)

Yield: 90%, m.p.: 175-176 ℃, ^{1}H NMR (400 MHz, Chloroform-*d*) δ 7.45 (d, J = 8.3 Hz, 2H), 7.39 (s, 1H), 7.34 (t, J = 7.2 Hz, 2H), 7.30–7.22 (m, 3H), 7.13 (s, 1H), 7.11 (s, 1H), 5.87 (d, J = 17.0 Hz, 1H), 5.23 (d, J = 17.8 Hz, 1H), 4.70 (s, 1H), 2.58–2.10 (m, 2H), 1.83–1.69 (m, 2H), 1.65–1.43 (m, 4H). ^{13}C NMR (101

MHz, Chloroform-*d*) δ 177.3, 140.5, 137.9, 132.0, 130.5, 128.9, 128.6, 127.2, 126.4, 122.3, 113.1, 77.4, 77.1, 76.7, 58.5, 50.9, 26.6, 25.6, 22.7, 21.2. Enantiomeric excess: 87%, determined by HPLC (Daicel Chirapak AD-H, hexane/*i*-PrOH = 70/ 30, flow rate 1.0 mL/min): T_R = 10.002 min (minor), T_R = 14.087 min (major).

(*R*)-1-Benzyl-4-(4-tolyl)-3,4,5,6,7,8-hexahydroquinazoline-2(1*H*)-thione (5e)

Yield: 88%, m.p.: 130-131 ℃, ^{1}H NMR (400 MHz, Chloroform-*d*) δ 7.37–7.31 (m, 2H), 7.31–7.24 (m, 4H), 7.14 (s, 4H), 5.86 (d, *J* = 14.5 Hz, 1H), 5.27 (d, *J* = 8.6 Hz, 1H), 4.71 (s, 1H), 2.35 (s, 3H), 2.21 (s, 2H), 1.89–1.72 (m, 2H), 1.66–1.38 (m, 4H). ^{13}C NMR (101 MHz, Chloroform-*d*) δ 177.3, 138.6, 138.1, 129.9, 129.5, 128.6, 127.1, 127.0, 126.4, 113.6, 59.0, 51.0, 26.6, 25.6, 22.8, 21.2. Enantiomeric excess: 80%, determined by HPLC (Daicel Chirapak AD-H, hexane/*i*-PrOH = 70/ 30, flow rate 1.5 mL/min): T_R = 3.278 min (minor), T_R = 7.154 min (major).

(*R*)-1-Benzyl-4-(4-methoxyphenyl)-3,4,5,6,7,8-hexahydroquinazoline-2(1*H*)-thione (5f)

Yield: 85%, m.p.: 142-145 ℃, ^{1}H NMR (400 MHz, Chloroform-*d*) δ 7.37 – 7.31 (m, 2H), 7.29–7.24 (m, 3H), 7.21–7.12 (m, 2H), 7.02 (s, 1H), 6.86 (d, *J* = 8.6 Hz, 2H), 5.83 (d, *J* = 8.5 Hz, 1H), 5.31 (d, *J* = 10.0 Hz, 1H), 4.70 (s, 1H), 3.80 (s, 3H), 2.21 (d, *J* = 5.9 Hz, 2H), 1.83–1.71 (m, 2H), 1.63–1.37 (m, 4H). ^{13}C NMR (101 MHz, Chloroform-*d*) δ 177.3, 159.6, 138.1, 133.7, 129.9, 128.6,

128.4, 127.1, 126.4, 58.6, 55.4, 50.9, 26.6, 25.6, 22.8, 21.2. Enantiomeric excess: 83%, determined by HPLC (Daicel Chirapak AD-H, hexane/*i*-PrOH = 70/ 30, flow rate 1.0 mL/min): T_R = 8.528 min (minor), T_R = 9.287 min (major).

(*R*)-1-Benzyl-4-(4-fluorophenyl)-3,4,5,6,7,8-hexahydroquinazoline-2(1*H*)-thione (5g)

Yield: 89%, m.p.: 172-175 ℃, ^{1}H NMR (400 MHz, Chloroform-*d*) δ 7.50 (s, 1H), 7.39–7.31 (m, 2H), 7.32–7.24 (m, 3H), 7.26–7.17 (m, 2H), 7.01 (t, *J* = 8.6 Hz, 2H), 5.89 (d, *J* = 9.3 Hz, 1H), 5.25 (d, *J* = 8.5 Hz, 1H), 4.73 (s, 1H), 2.35–2.13 (m, 2H), 1.93–1.70 (m, 2H), 1.68–1.40 (m, 4H). ^{13}C NMR (151 MHz, Chloroform-*d*) δ 175.9, 162.3, 160.7, 136.9, 136.2, 129.3, 127.9, 127.6, 126.1, 125.4, 114.8, 112.3, 57.3, 49.8, 25.6, 24.5, 21.7, 20.1. Anal. calc. for $C_{21}H_{21}N_2FS$: C 71.56, H 6.01; found: C 71.48, H 5.96. Enantiomeric excess: 73%, determined by HPLC (Daicel Chirapak AD-H, hexane/*i*-PrOH = 70/ 30, flow rate 1.5 mL/min): T_R = 6.904 min (minor), T_R = 13.005 min (major).

(*R*)-1-Benzyl-4-(4-nitrophenyl)-3,4,5,6,7,8-hexahydroquinazoline-2(1*H*)-thione (5h)

Yield: 79%, m.p.: 170-175 ℃, ^{1}H NMR (400 MHz, Chloroform-*d*) δ 8.17 (d, *J* = 8.5 Hz, 2H), 7.72 (s, 1H), 7.41 (d, *J* = 8.5 Hz, 2H), 7.32 (dd, *J* = 13.1, 7.1 Hz, 3H), 7.28–7.23 (m, 2H), 5.88 (d, *J* = 12.0 Hz, 1H), 5.21 (d, *J* = 17.6 Hz, 1H), 4.83 (s, 1H), 2.37–2.18 (m, 2H), 1.96–1.71 (m, 2H), 1.69–1.41 (m, 4H). ^{13}C NMR (101 MHz, Chloroform-*d*) δ 177.6, 148.3, 147.8, 137.7, 131.2, 128.7, 128.0, 127.3, 126.5, 124.2, 123.5, 112.5, 58.5, 50.9, 26.8, 25.7, 22.6, 21.1.

Enantiomeric excess: 42%, determined by HPLC (Daicel Chirapak AD-H, hexane/*i*-PrOH = 70/ 30, flow rate 1.0 mL/min): T_R = 12.190 min (minor), T_R = 23.253 min (major).

(*R*)-1-Benzyl-4-(2-nitrophenyl)-3,4,5,6,7,8-hexahydroquinazoline-2(1*H*)-thione (5i)

Yield: 76%, m.p.: 182-185 ℃, ^{1}H NMR (400 MHz, Chloroform-*d*) δ 8.02 (d, *J* = 8.0 Hz, 1H), 7.60–7.49 (m, 2H), 7.45 (s, 1H), 7.34–7.26 (m, 6H), 6.19 (d, *J* = 6.5 Hz, 1H), 5.18 (s, 1H), 5.01 (d, *J* = 18.5 Hz, 1H), 2.36 (s, 2H), 1.93–1.78 (m, 1H), 1.75–1.58 (m, 4H), 1.47–1.36 (m, 1H). ^{13}C NMR (101 MHz, Chloroform-*d*) δ 177.6, 149.0, 137.8, 134.4, 133.4, 129.7, 129.5, 128.6, 127.4, 126.9, 125.4, 111.5, 53.6, 50.9, 26.8, 25.8, 22.7, 21.1. Anal. calc. for $C_{21}H_{21}N_3O_2S$: C 66.47, H 5.58; found: C 66.36, H 5.49. Enantiomeric excess: 23%, determined by HPLC (Daicel Chirapak AD-H, hexane/*i*-PrOH = 70/ 30, flow rate 1.5 mL/min): T_R = 5.086 min (minor), T_R= 6.648 min (major).

(*R*)-1-Benzyl-4-(2-chlorophenyl)-3,4,5,6,7,8-hexahydroquinazoline-2(1*H*)-thione (5j)

Yield: 72%, m.p.: 190-191 ℃, ^{1}H NMR (400 MHz, Chloroform-*d*) δ 7.41 (dd, *J* = 7.7, 1.4 Hz, 1H), 7.36–7.27 (m, 5H), 7.25–7.21 (m, 2H), 7.17 (dd, *J* = 7.5, 1.8 Hz, 1H), 7.05 (s, 1H), 6.07 (d, *J* = 11.3 Hz, 1H), 5.23 (s, 1H), 5.10 (d, *J* = 13.7 Hz, 1H), 2.35–2.25 (m, 2H), 2.00–1.75 (m, 2H), 1.73–1.55 (m, 3H), 1.52–1.40 (m, 1H). ^{13}C NMR (101 MHz, Chloroform-*d*) δ 177.7, 137.8, 137.3, 133.5, 132.0, 130.0, 129.8, 128.9, 128.6, 127.6, 127.2, 126.6, 112.4, 54.9, 51.0, 26.6, 25.7,

22.8, 21.2. Anal. calc. for $C_{21}H_{21}ClN_2S$: C 68.37, H 5.74; found: C 68.28, H 5.69. Enantiomeric excess: 53%, determined by HPLC (Daicel Chirapak AD-H, hexane/*i*-PrOH = 70/ 30, flow rate 1.5 mL/min): T_R = 6.389 min (minor), T_R= 11.041 min (major).

(*R*)-1-Benzyl-4-(4-(*tert*-butyl)phenyl)-3,4,5,6,7,8-hexahydroquinazo-line-2(1*H*)-thione (5k)

Yield: 73%, m.p.: 179-180 ℃, ^{1}H NMR (400 MHz, Chloroform-*d*) δ 7.39–7.29 (m, 4H), 7.28–7.24 (m, 3H), 7.22–7.15 (m, 2H), 6.94 (s, 1H), 5.86 (d, *J* = 9.6 Hz, 1H), 5.30 (d, *J* = 14.3 Hz, 1H), 4.72 (s, 1H), 2.21 (d, *J* = 4.5 Hz, 2H), 1.81 (q, *J* = 6.1 Hz, 2H), 1.64–1.41 (m, 4H), 1.32 (s, 9H). ^{13}C NMR (101 MHz, Chloroform-*d*) δ 177.4, 151.3, 138.4, 138.1, 130.0, 128.6, 127.0, 126.9, 126.4, 125.8, 113.6, 58.9, 51.0, 34.6, 31.3, 26.7, 25.6, 22.8, 21.2. Enantiomeric excess: 20%, determined by HPLC (Daicel Chirapak AD-H, hexane/*i*-PrOH = 70/ 30, flow rate 1.0 mL/min): T_R = 6,262 min (minor), T_R = 10.247 min (major).

II NMR Spectra

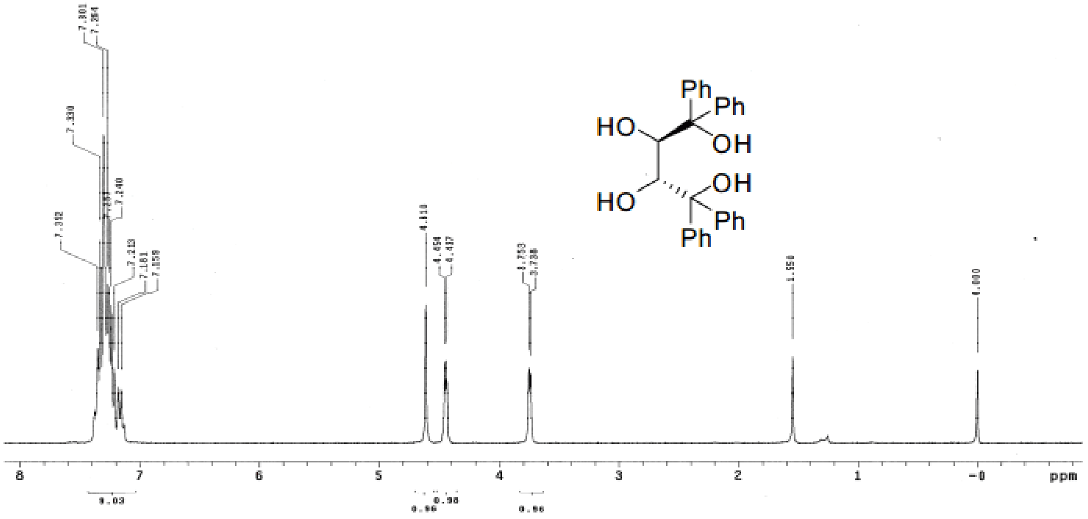

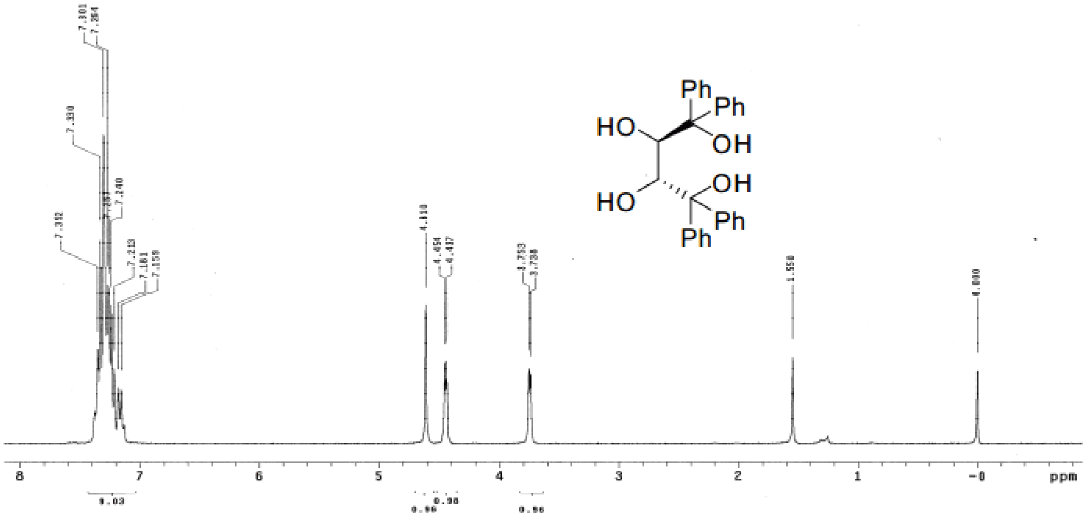

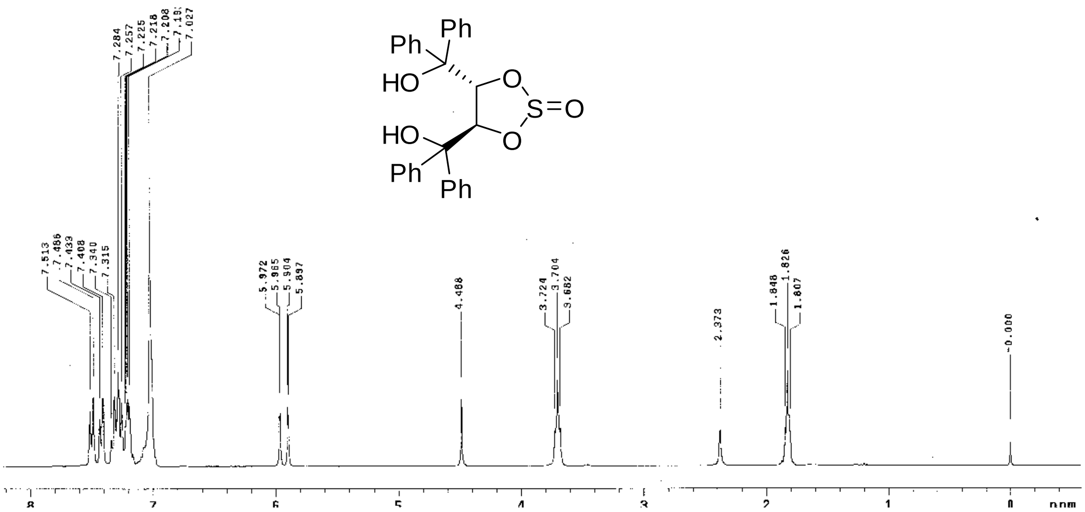
Ph
Ph
HO
O
S=O
HO
O
Ph
Ph
7.513
7.486
7.433
7.408
7.340
7.315
7.284
7.257
7.225
7.218
7.208
7.027
5.972
5.965
5.904
5.897
4.488
3.724
3.704
3.682
2.373
1.848
1.826
1.807
0.000
8
7
6
5
4
3
2
1
0
ppm

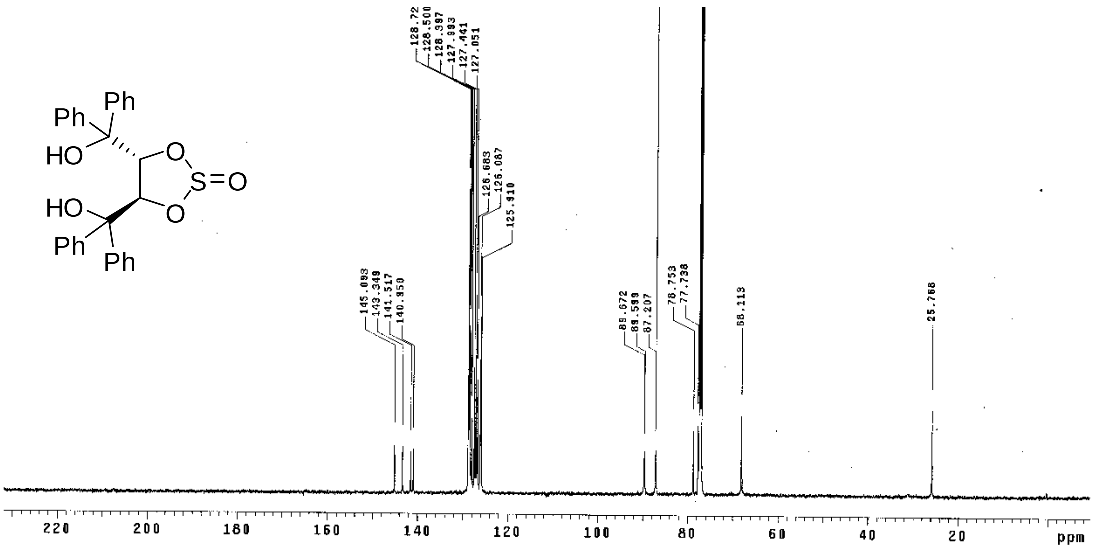
Ph
Ph
HO
O
S=O
HO
O
Ph
Ph
128.72
128.500
128.397
127.993
127.441
127.051
126.683
126.087
125.910
145.093
143.349
141.517
140.950
89.672
89.589
87.207
78.753
77.738
68.113
25.768
220
200
180
160
140
120
100
80
60
40
20
ppm

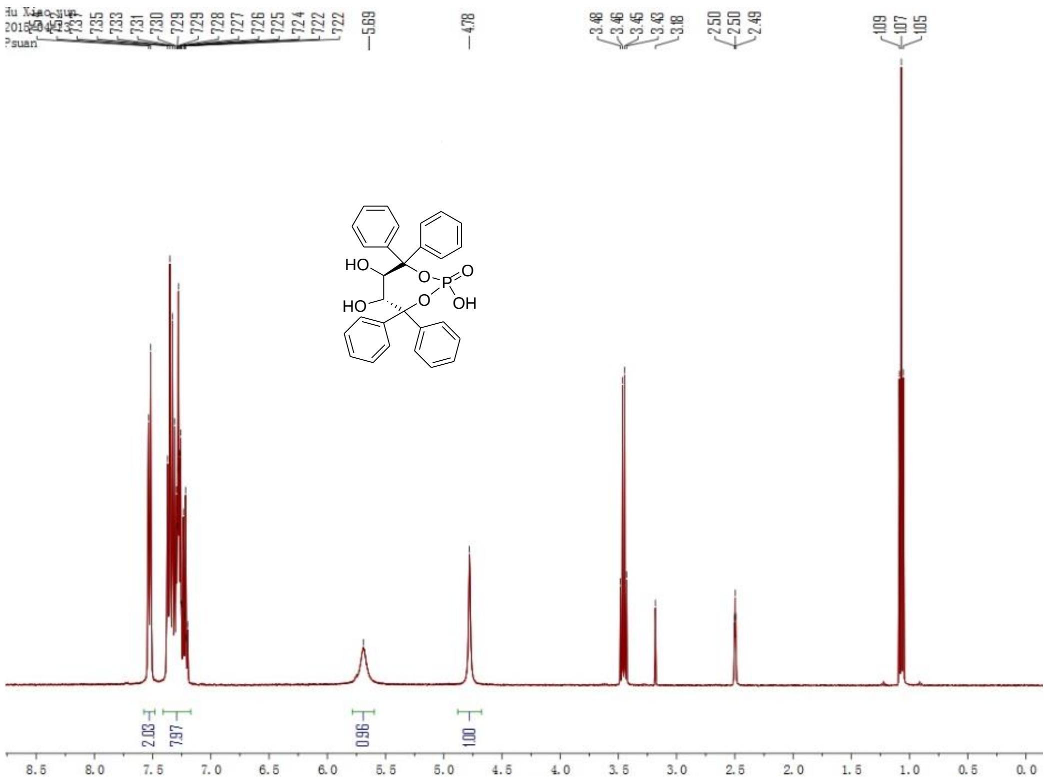

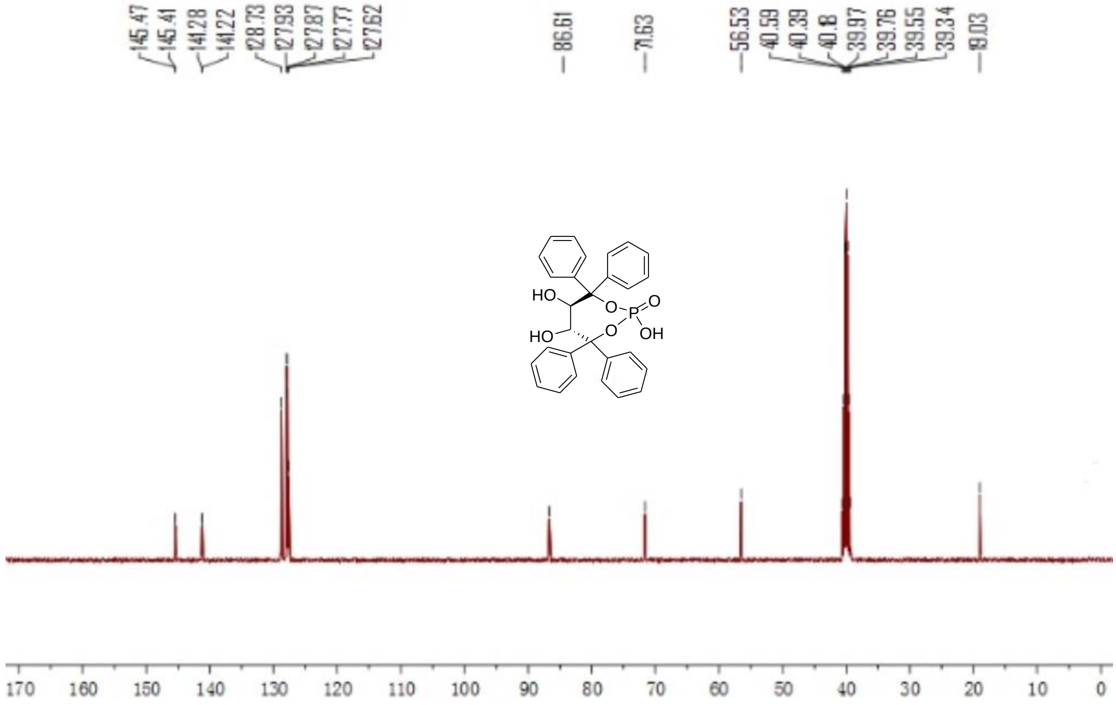

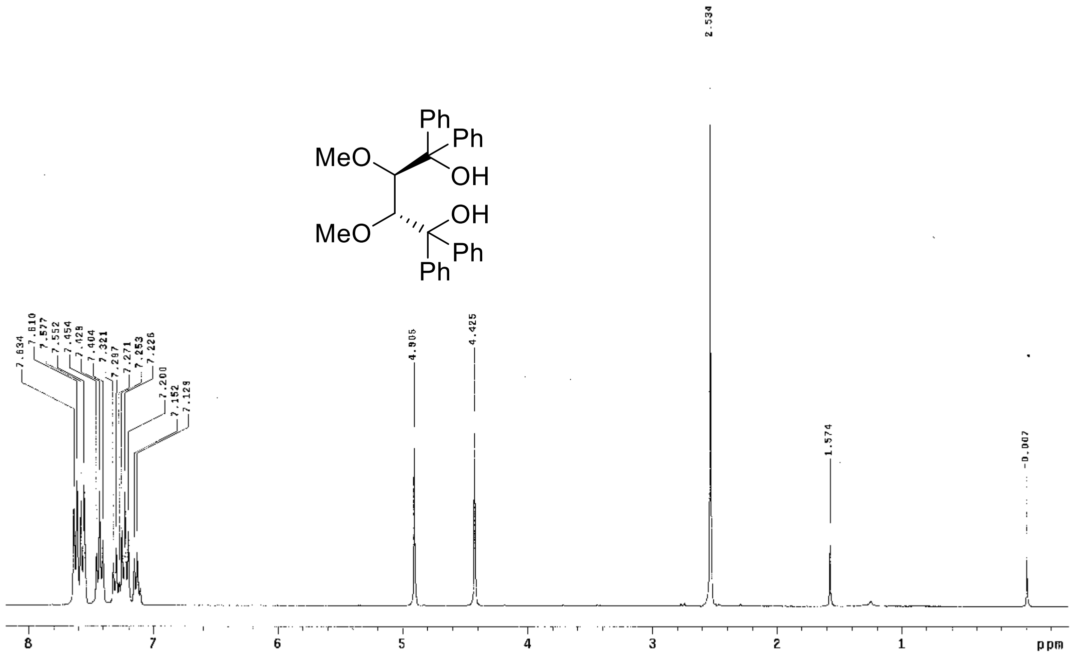
Ph
Ph
MeO
OH
MeO
OH
Ph
Ph
ppm

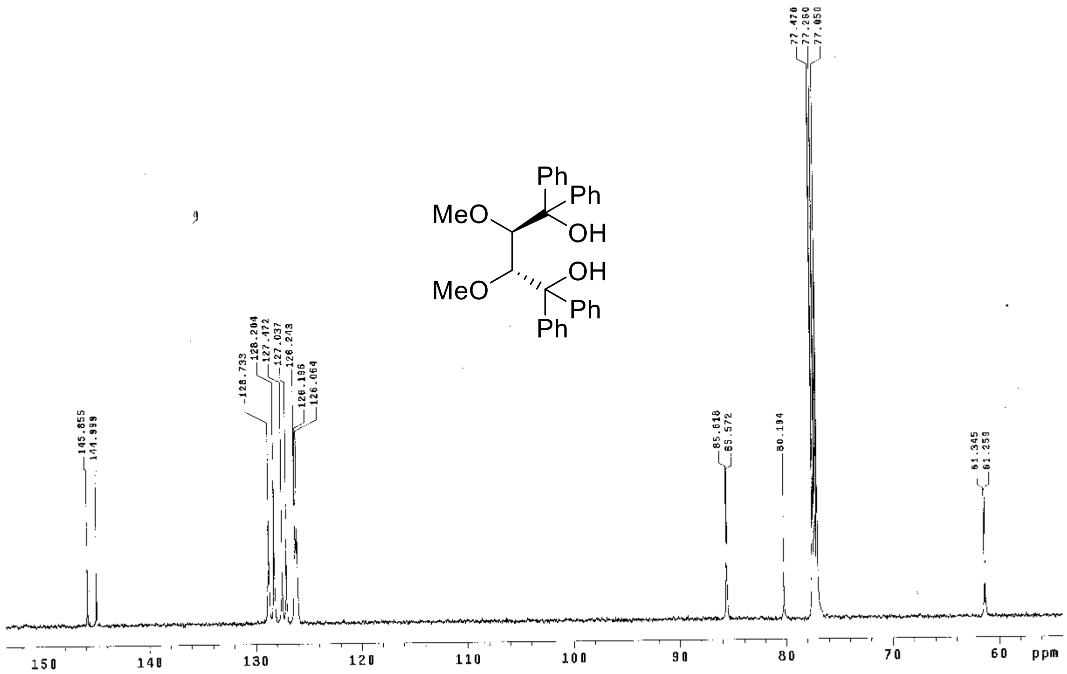
Ph
Ph
MeO
OH
MeO
OH
Ph
Ph
ppm

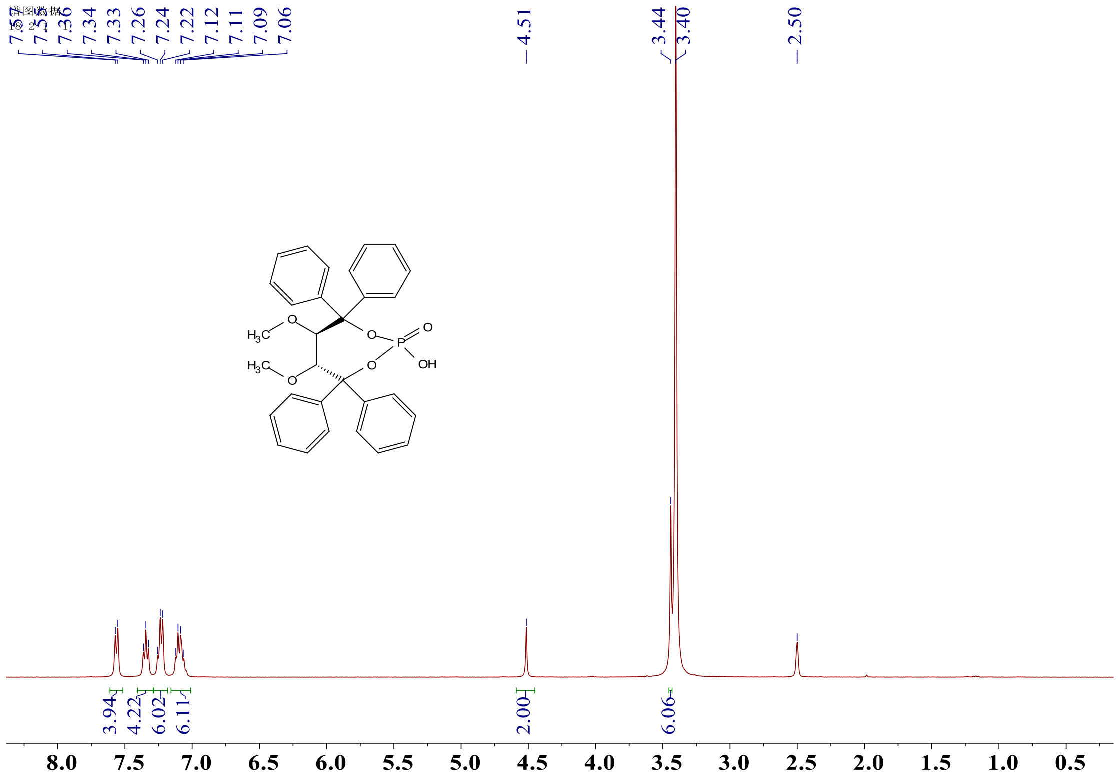
7.36
7.34
7.33
7.26
7.24
7.22
7.12
7.11
7.09
7.06
4.51
3.44
3.40
2.50
H3C
O
O
P
O
OH
3.94
4.22
6.02
6.11
2.00
6.06
8.0 7.5 7.0 6.5 6.0 5.5 5.0 4.5 4.0 3.5 3.0 2.5 2.0 1.5 1.0 0.5

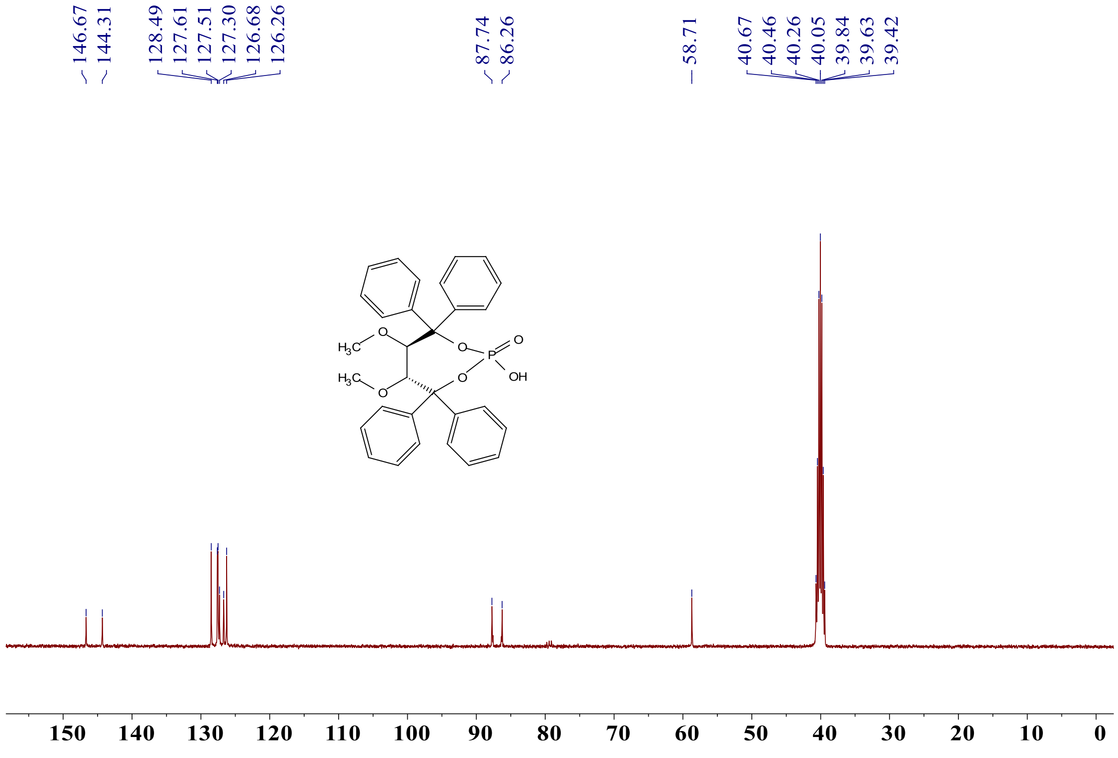
146.67
144.31
128.49
127.61
127.51
127.30
126.68
126.26
87.74
86.26
58.71
40.67
40.46
40.26
40.05
39.84
39.63
39.42
H3C
O
O
P
O
OH
150 140 130 120 110 100 90 80 70 60 50 40 30 20 10 0

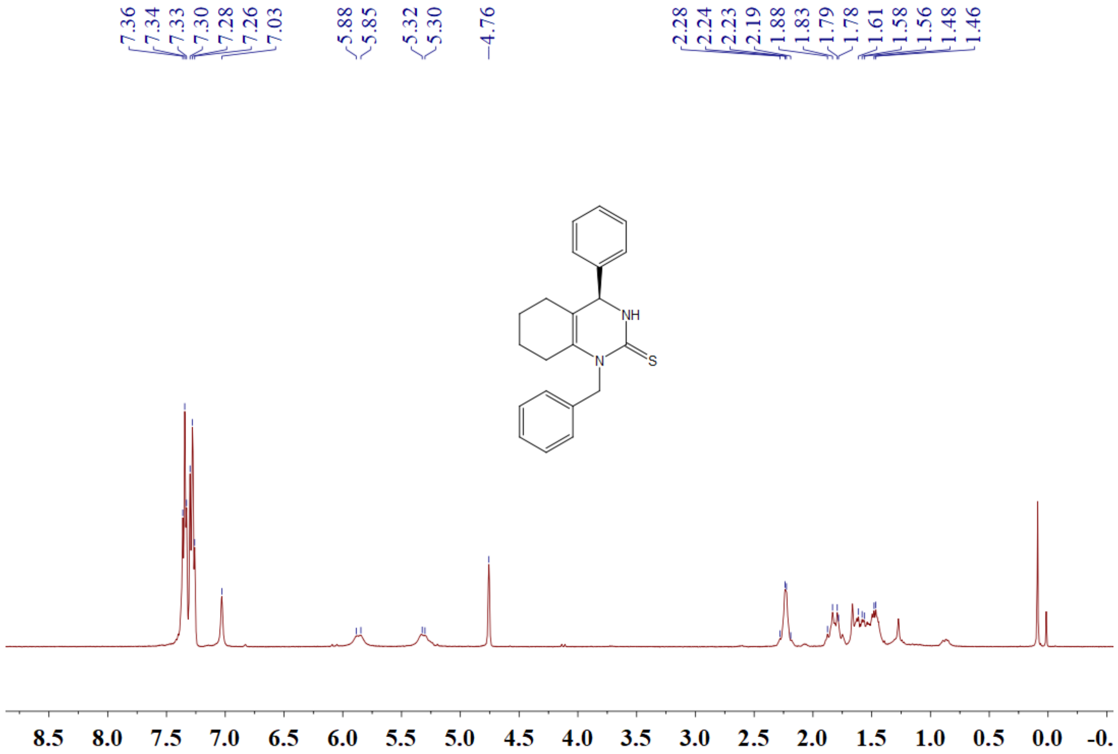
7.36 7.34 7.33 7.30 7.28 7.26 7.03
5.88 5.85
5.32 5.30
4.76
2.28 2.24 2.23 2.19 1.88 1.83 1.79 1.78 1.61 1.58 1.56 1.48 1.46
NH
N
S
8.5 8.0 7.5 7.0 6.5 6.0 5.5 5.0 4.5 4.0 3.5 3.0 2.5 2.0 1.5 1.0 0.5 0.0 -0.

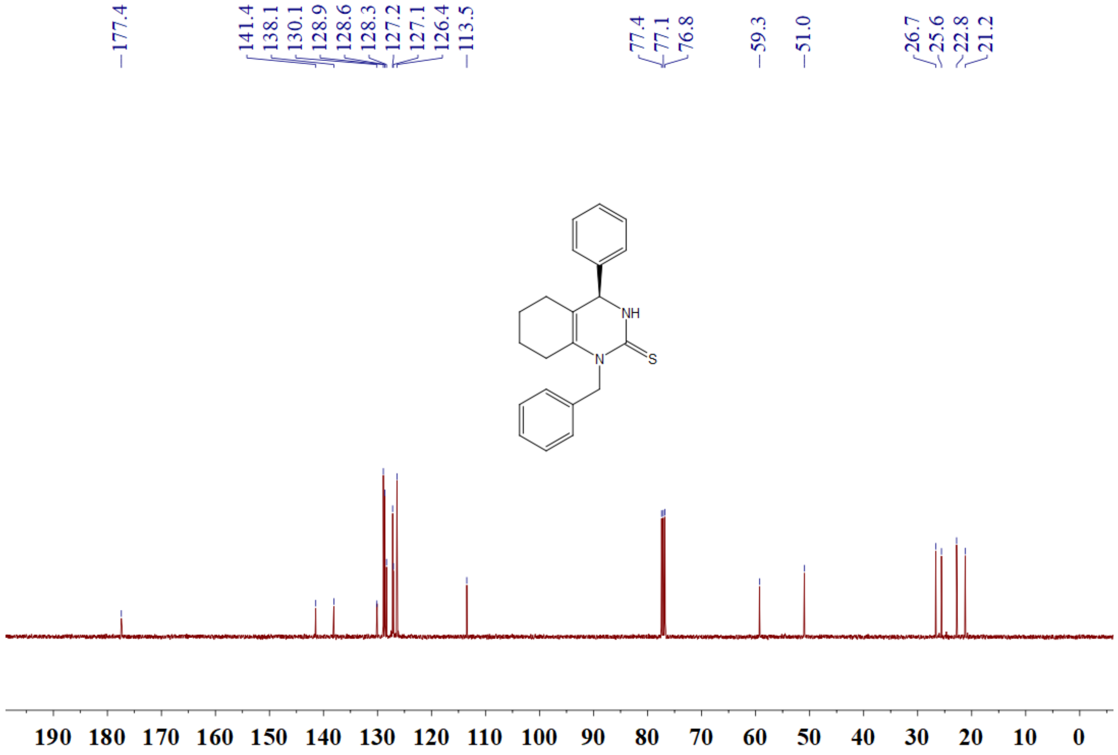
177.4
141.4 138.1 130.1 128.9 128.6 128.3 127.2 127.1 126.4 113.5
77.4 77.1 76.8
59.3
51.0
26.7 25.6 22.8 21.2
NH
N
S
190 180 170 160 150 140 130 120 110 100 90 80 70 60 50 40 30 20 10 0

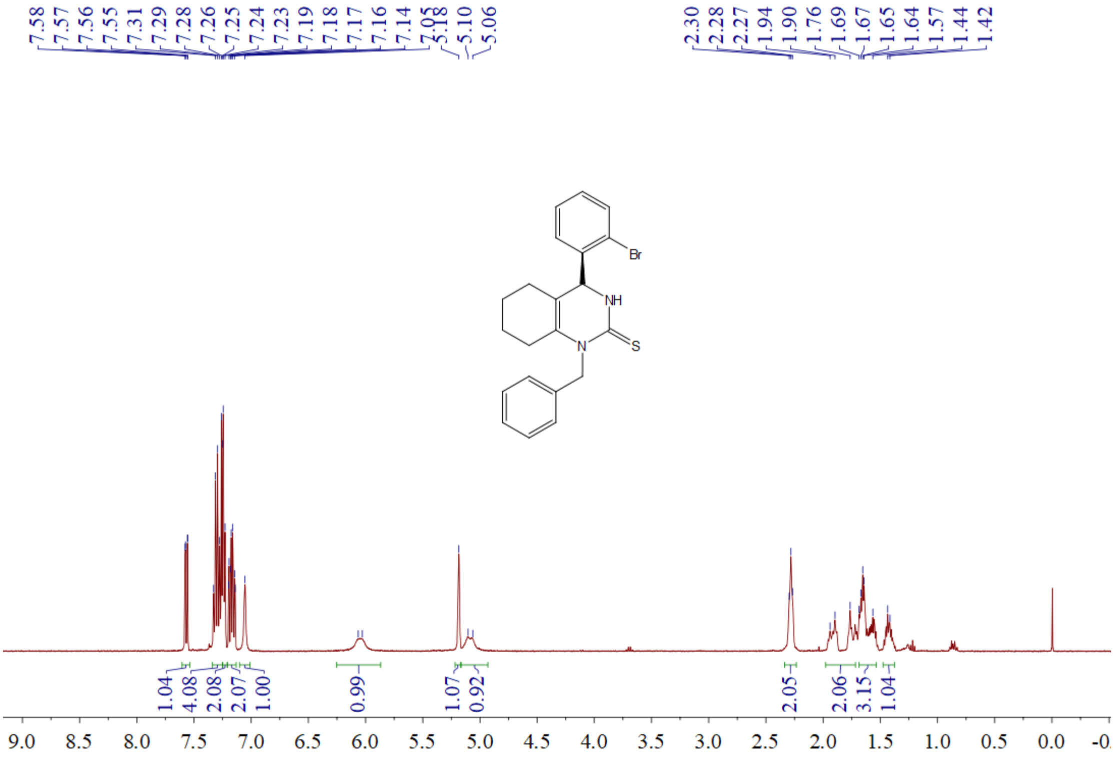
7.58
7.57
7.56
7.55
7.31
7.29
7.28
7.26
7.25
7.24
7.23
7.19
7.18
7.17
7.16
7.14
7.05
5.18
5.10
5.06
2.30
2.28
2.27
1.94
1.90
1.76
1.69
1.67
1.65
1.64
1.57
1.44
1.42
Br
NH
N
S
1.04
4.08
2.08
2.07
1.00
0.99
1.07
0.92
2.05
2.06
3.15
1.04
9.0 8.5 8.0 7.5 7.0 6.5 6.0 5.5 5.0 4.5 4.0 3.5 3.0 2.5 2.0 1.5 1.0 0.5 0.0 -0.

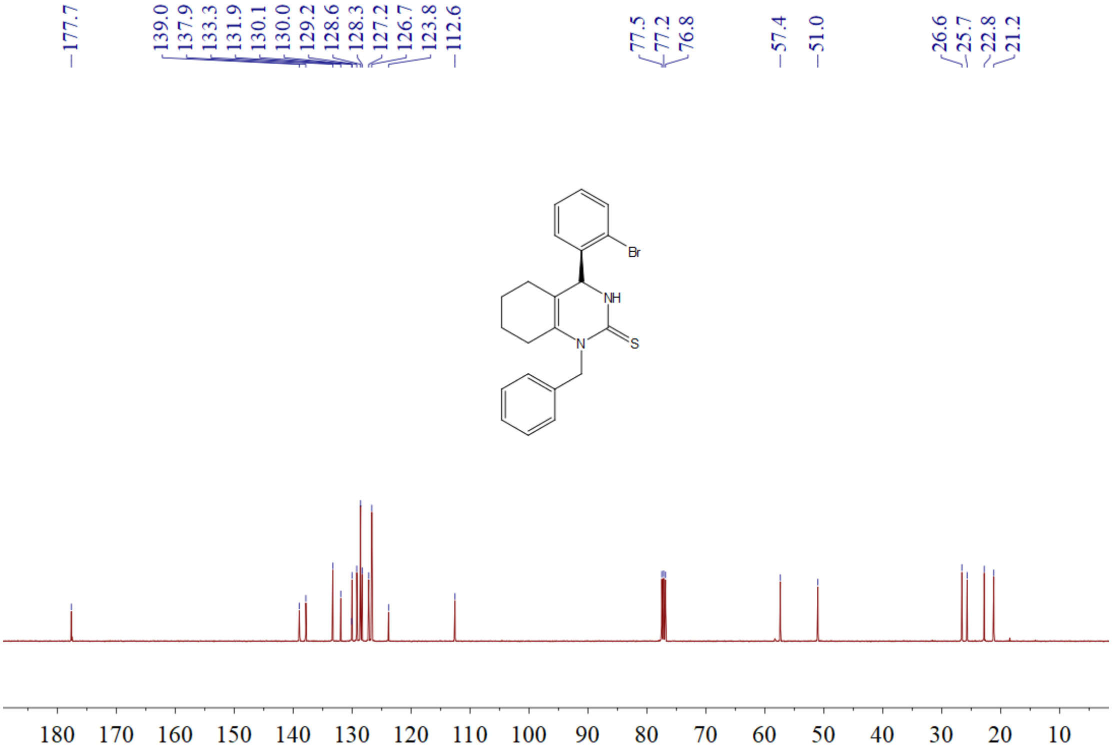
177.7
139.0
137.9
133.3
131.9
130.1
130.0
129.2
128.6
128.3
127.2
126.7
123.8
112.6
77.5
77.2
76.8
57.4
51.0
26.6
25.7
22.8
21.2
Br
NH
N
S
180 170 160 150 140 130 120 110 100 90 80 70 60 50 40 30 20 10

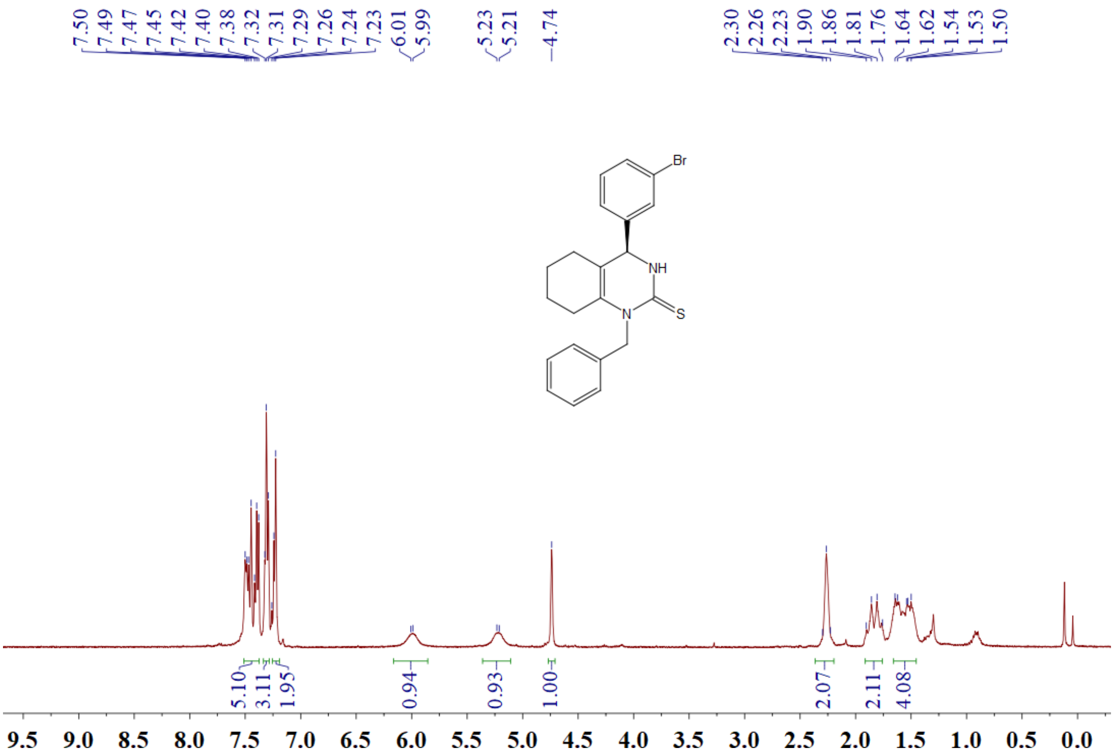
7.50
7.49
7.47
7.45
7.42
7.40
7.38
7.32
7.31
7.29
7.26
7.24
7.23
6.01
5.99
5.23
5.21
4.74
2.30
2.26
2.23
1.90
1.86
1.81
1.76
1.64
1.62
1.54
1.53
1.50
Br
NH
N
S
5.10
3.11
1.95
0.94
0.93
1.00
2.07
2.11
4.08
9.5 9.0 8.5 8.0 7.5 7.0 6.5 6.0 5.5 5.0 4.5 4.0 3.5 3.0 2.5 2.0 1.5 1.0 0.5 0.0

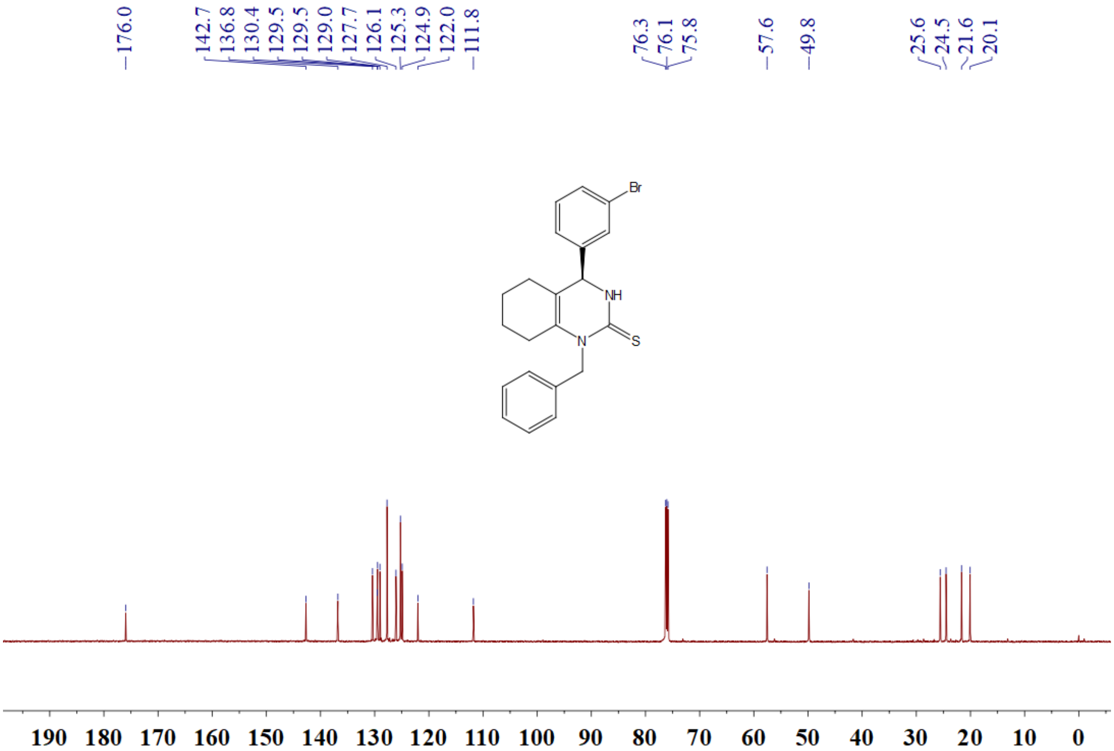
176.0
142.7
136.8
130.4
129.5
129.5
129.0
127.7
126.1
125.3
124.9
122.0
111.8
76.3
76.1
75.8
57.6
49.8
25.6
24.5
21.6
20.1
Br
NH
N
S
190 180 170 160 150 140 130 120 110 100 90 80 70 60 50 40 30 20 10 0

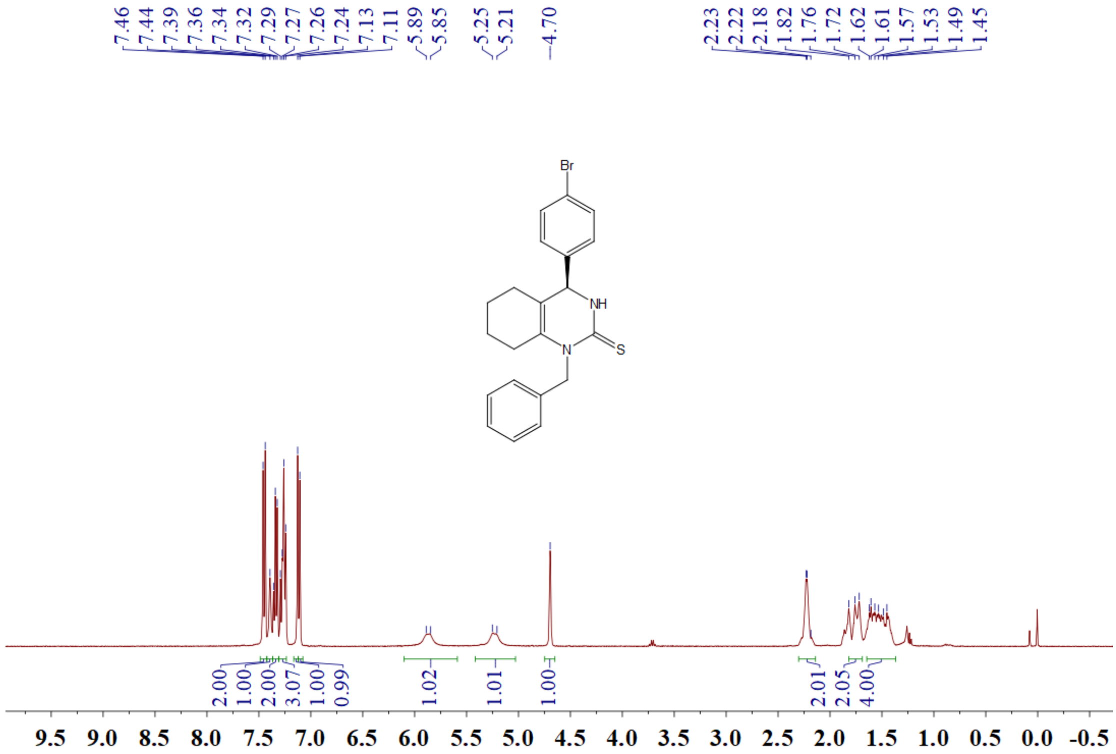
7.46
7.44
7.39
7.36
7.34
7.32
7.29
7.27
7.26
7.24
7.13
7.11
5.89
5.85
5.25
5.21
4.70
2.23
2.22
2.18
1.82
1.76
1.72
1.62
1.61
1.57
1.53
1.49
1.45
Br
NH
N
S
2.00
1.00
2.00
3.07
1.00
0.99
1.02
1.01
1.00
2.01
2.05
4.00
9.5 9.0 8.5 8.0 7.5 7.0 6.5 6.0 5.5 5.0 4.5 4.0 3.5 3.0 2.5 2.0 1.5 1.0 0.5 0.0 -0.5

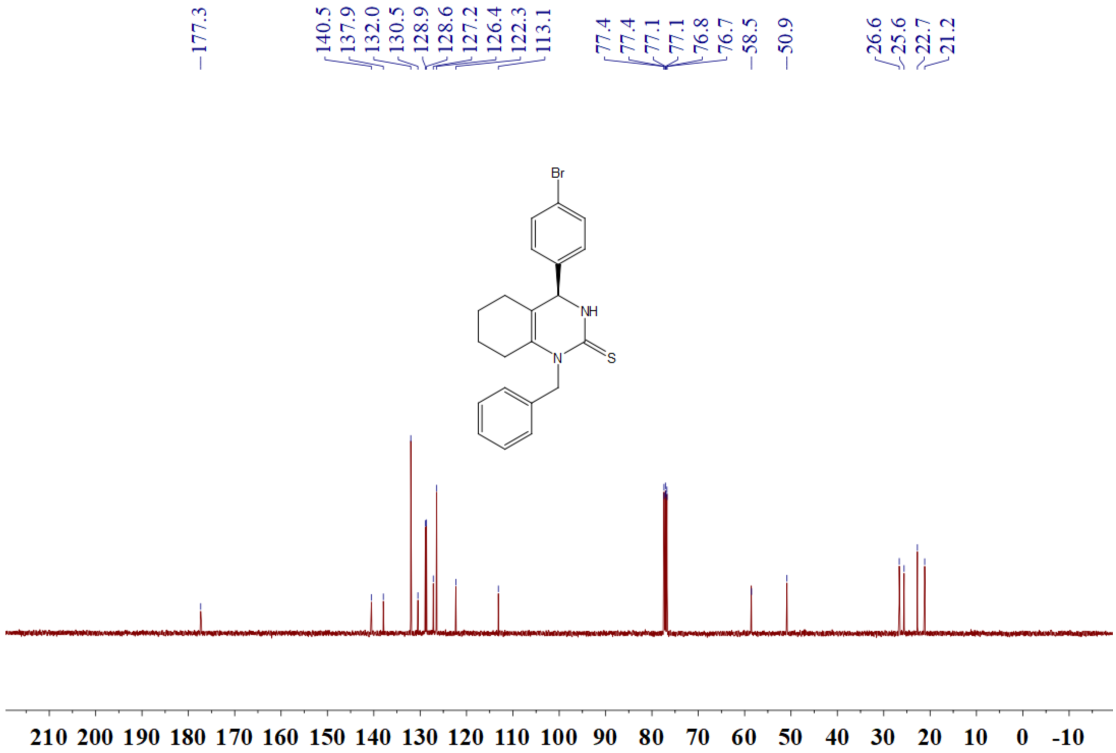
177.3
140.5
137.9
132.0
130.5
128.9
128.6
127.2
126.4
122.3
113.1
77.4
77.4
77.1
77.1
76.8
76.7
58.5
50.9
26.6
25.6
22.7
21.2
Br
NH
N
S
210 200 190 180 170 160 150 140 130 120 110 100 90 80 70 60 50 40 30 20 10 0 -10

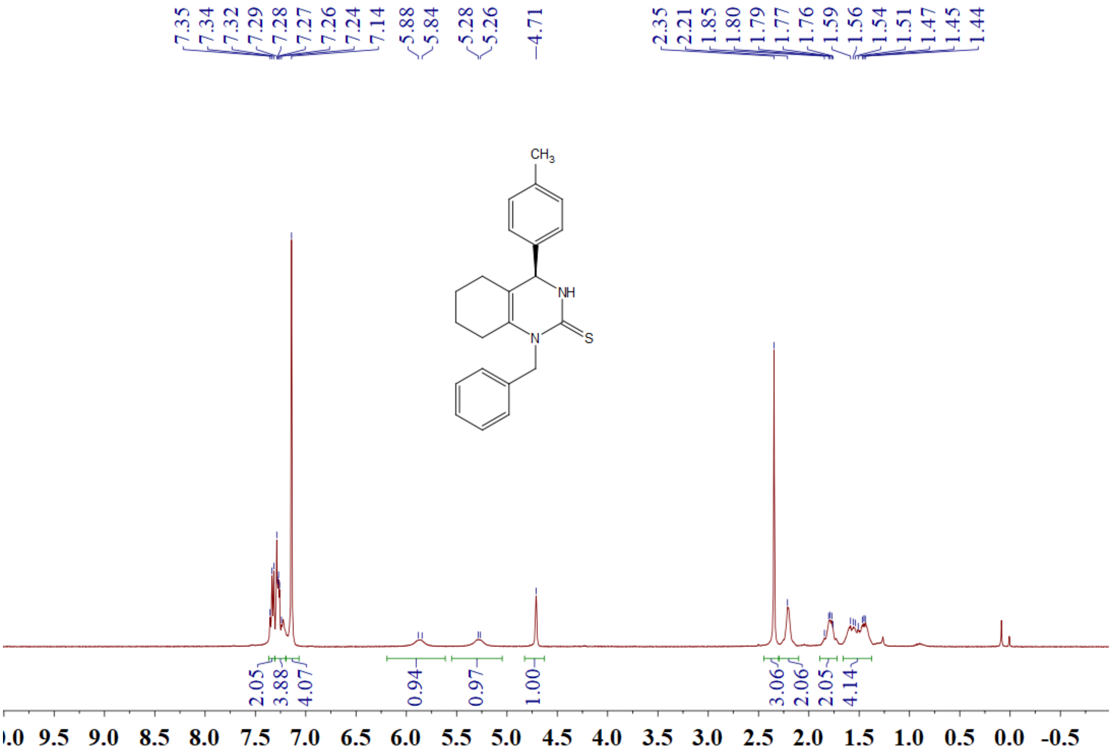
7.35 7.34 7.32 7.29 7.28 7.27 7.26 7.24 7.14
5.88 5.84
5.28 5.26
4.71
2.35 2.21 1.85 1.80 1.79 1.77 1.76 1.59 1.56 1.54 1.51 1.47 1.45 1.44
CH3
NH
S
N
2.05 3.88 4.07 0.94 0.97 1.00 3.06 2.06 2.05 4.14
.0 9.5 9.0 8.5 8.0 7.5 7.0 6.5 6.0 5.5 5.0 4.5 4.0 3.5 3.0 2.5 2.0 1.5 1.0 0.5 0.0 -0.5

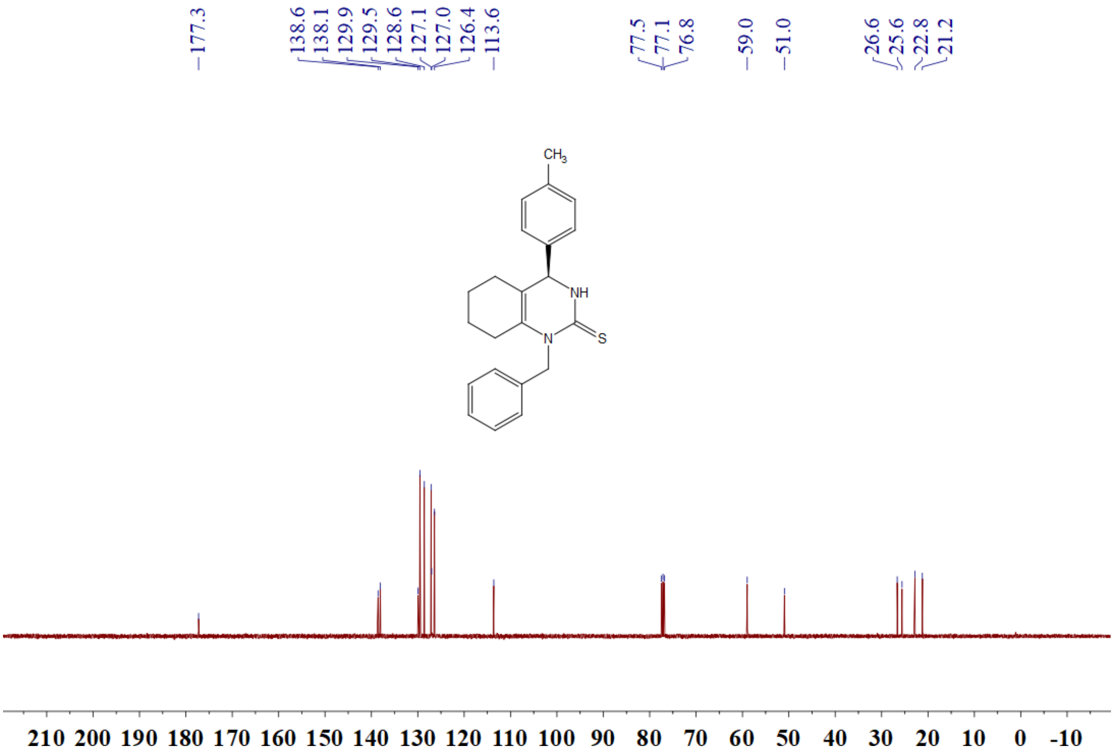
177.3
138.6 138.1 129.9 129.5 128.6 127.1 127.0 126.4 113.6
77.5 77.1 76.8
59.0
51.0
26.6 25.6 22.8 21.2
CH3
NH
S
N
210 200 190 180 170 160 150 140 130 120 110 100 90 80 70 60 50 40 30 20 10 0 -10

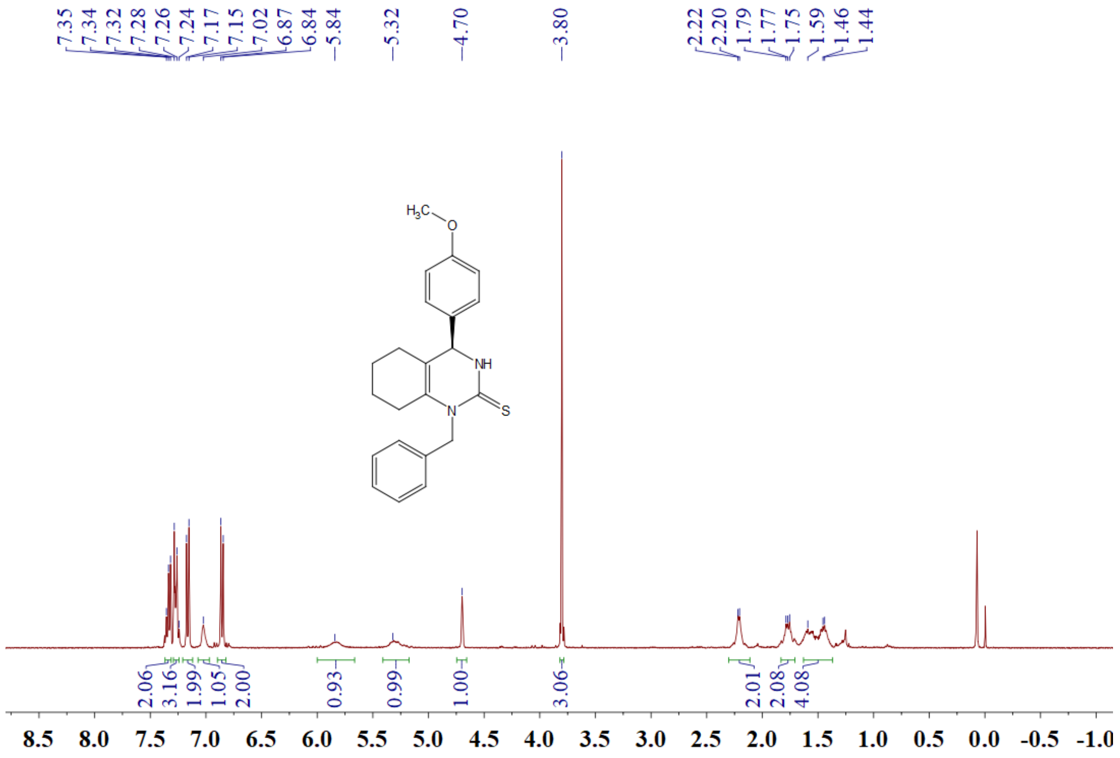
7.35 7.34 7.32 7.28 7.26 7.24 7.17 7.15 7.02 6.87 6.84 5.84 5.32 4.70 3.80 2.22 2.20 1.79 1.77 1.75 1.59 1.46 1.44
H_3C O NH N S
2.06 3.16 1.99 1.05 2.00 0.93 0.99 1.00 3.06 2.01 2.08 4.08
8.5 8.0 7.5 7.0 6.5 6.0 5.5 5.0 4.5 4.0 3.5 3.0 2.5 2.0 1.5 1.0 0.5 0.0 -0.5 -1.0

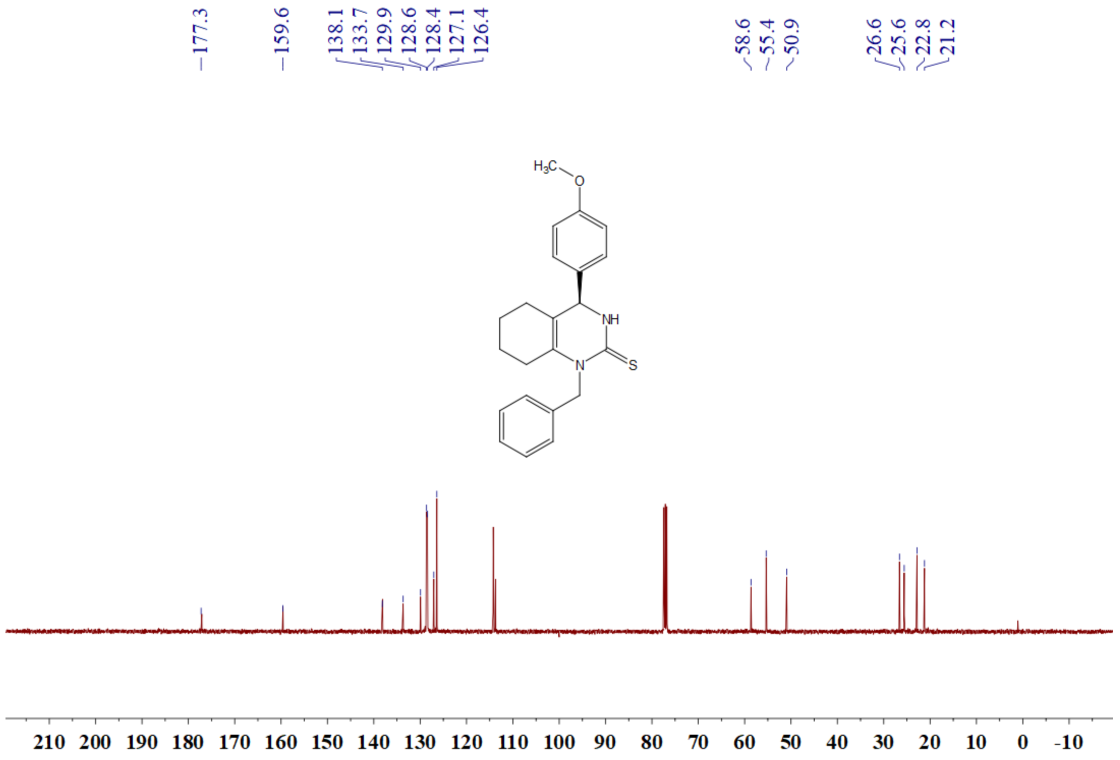
177.3 159.6 138.1 133.7 129.9 128.6 128.4 127.1 126.4 58.6 55.4 50.9 26.6 25.6 22.8 21.2
H_3C O NH N S
210 200 190 180 170 160 150 140 130 120 110 100 90 80 70 60 50 40 30 20 10 0 -10

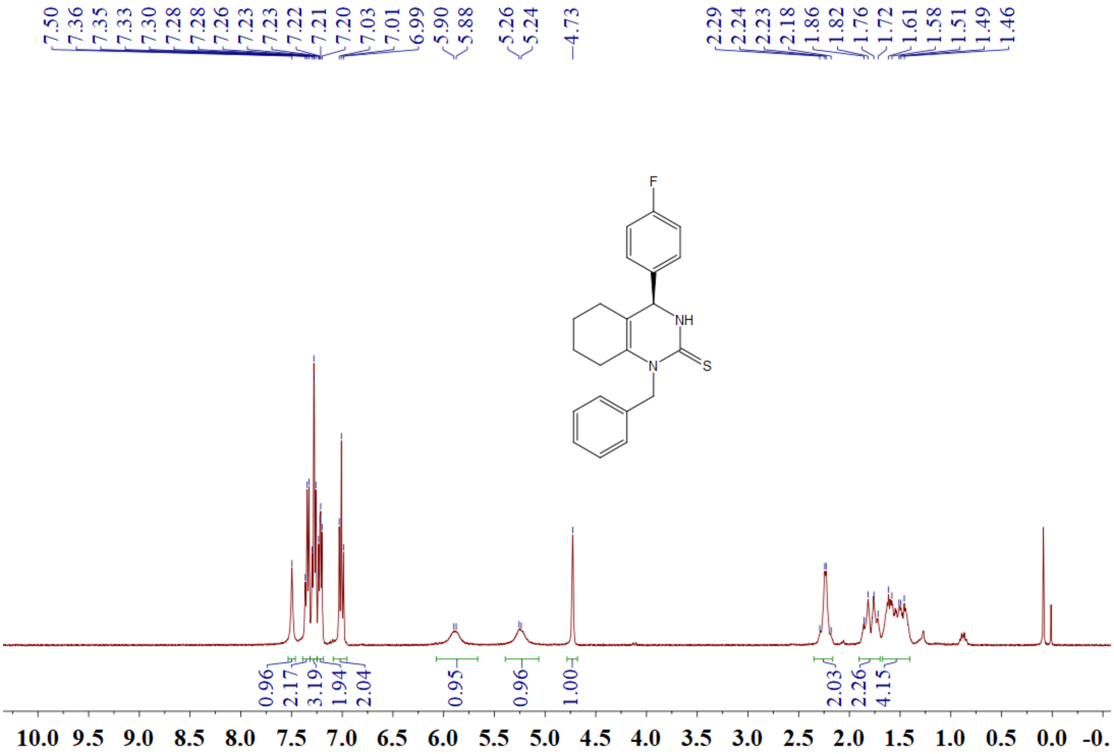
7.50
7.36
7.35
7.33
7.30
7.28
7.28
7.26
7.23
7.23
7.22
7.21
7.20
7.03
7.01
6.99
5.90
5.88
5.26
5.24
4.73
2.29
2.24
2.23
2.18
1.86
1.82
1.76
1.72
1.61
1.58
1.51
1.49
1.46
F
NH
N
S
0.96
2.17
3.19
1.94
2.04
0.95
0.96
1.00
2.03
2.26
4.15
10.0 9.5 9.0 8.5 8.0 7.5 7.0 6.5 6.0 5.5 5.0 4.5 4.0 3.5 3.0 2.5 2.0 1.5 1.0 0.5 0.0

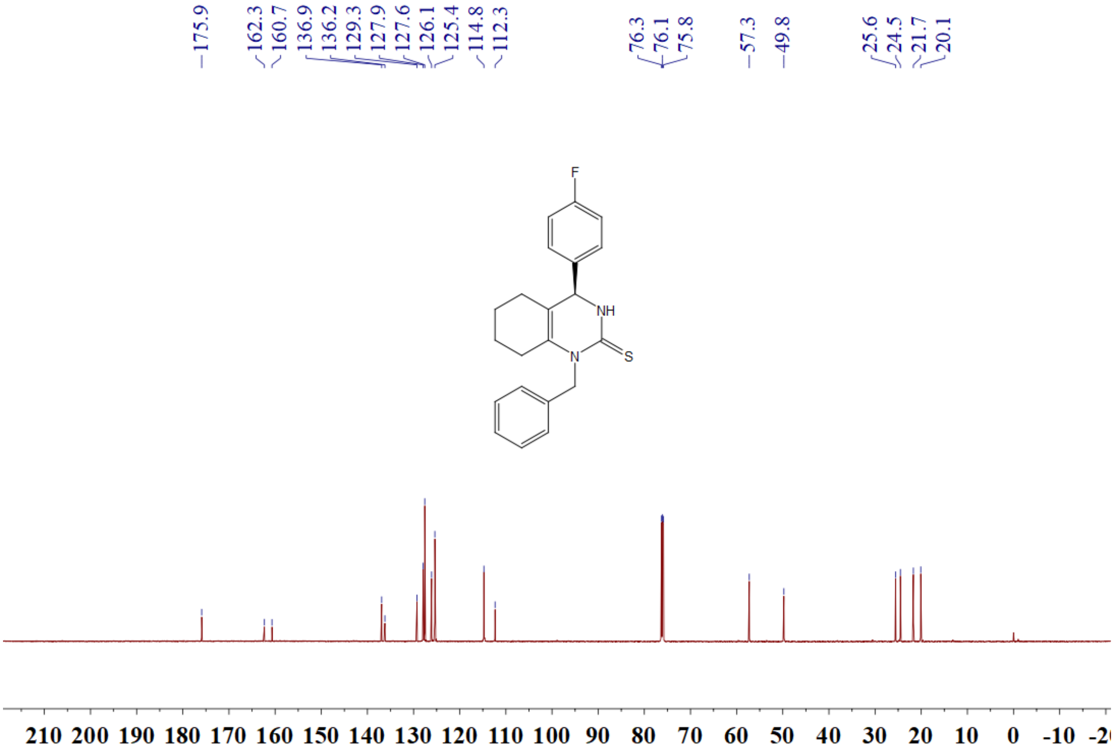
175.9
162.3
160.7
136.9
136.2
129.3
127.9
127.6
126.1
125.4
114.8
112.3
76.3
76.1
75.8
57.3
49.8
25.6
24.5
21.7
20.1
F
NH
N
S
210 200 190 180 170 160 150 140 130 120 110 100 90 80 70 60 50 40 30 20 10 0 -10

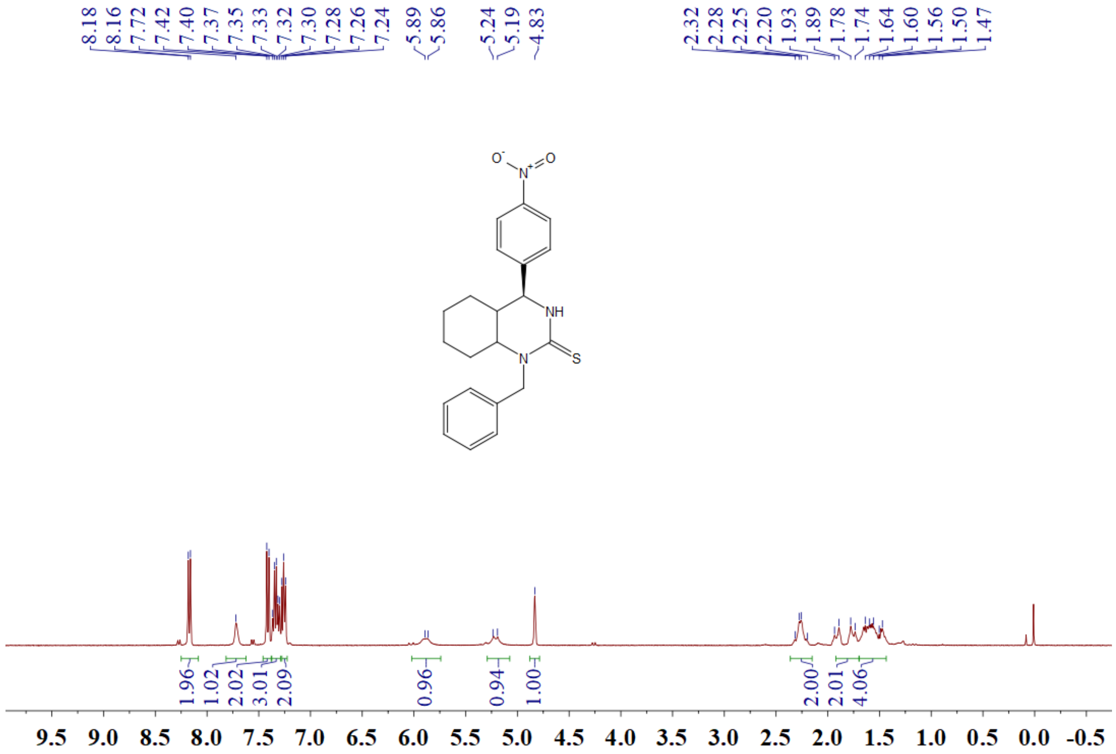
8.18
8.16
7.72
7.42
7.40
7.37
7.35
7.33
7.32
7.30
7.28
7.26
7.24
5.89
5.86
5.24
5.19
4.83
2.32
2.28
2.25
2.20
1.93
1.89
1.78
1.74
1.64
1.60
1.56
1.50
1.47
1.96
1.02
2.02
3.01
2.09
0.96
0.94
1.00
2.00
2.01
4.06
9.5 9.0 8.5 8.0 7.5 7.0 6.5 6.0 5.5 5.0 4.5 4.0 3.5 3.0 2.5 2.0 1.5 1.0 0.5 0.0 -0.5

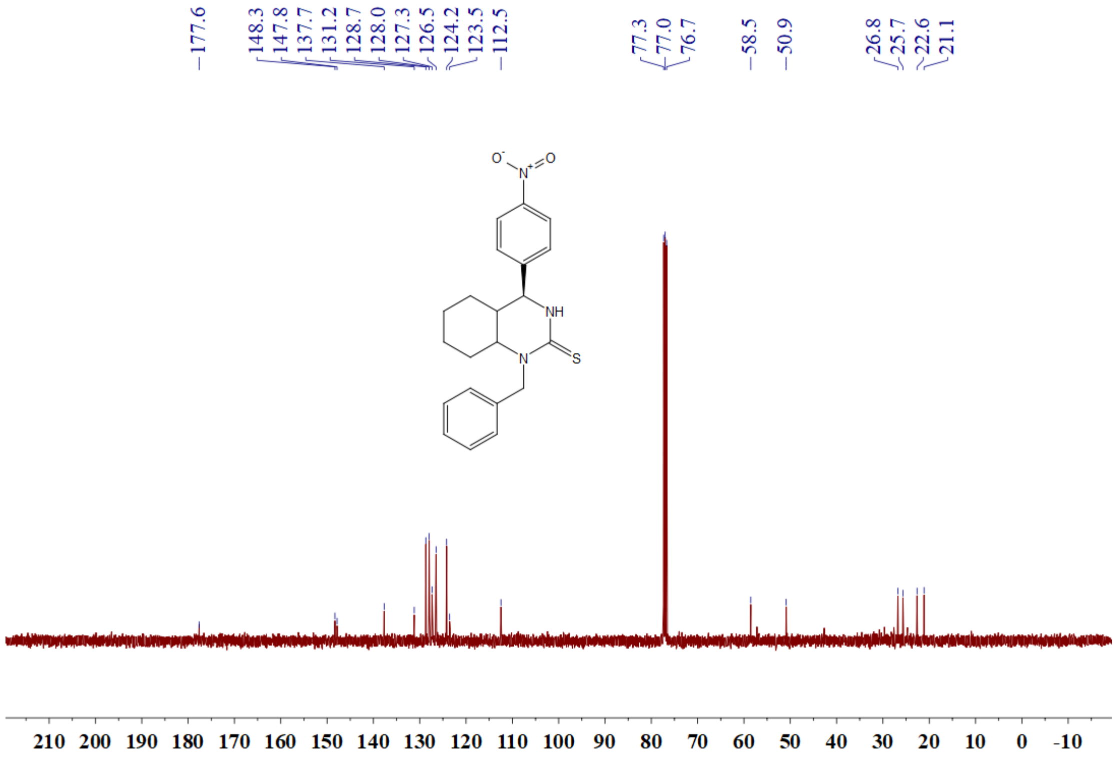
177.6
148.3
147.8
137.7
131.2
128.7
128.0
127.3
126.5
124.2
123.5
112.5
77.3
77.0
76.7
58.5
50.9
26.8
25.7
22.6
21.1
210 200 190 180 170 160 150 140 130 120 110 100 90 80 70 60 50 40 30 20 10 0 -10

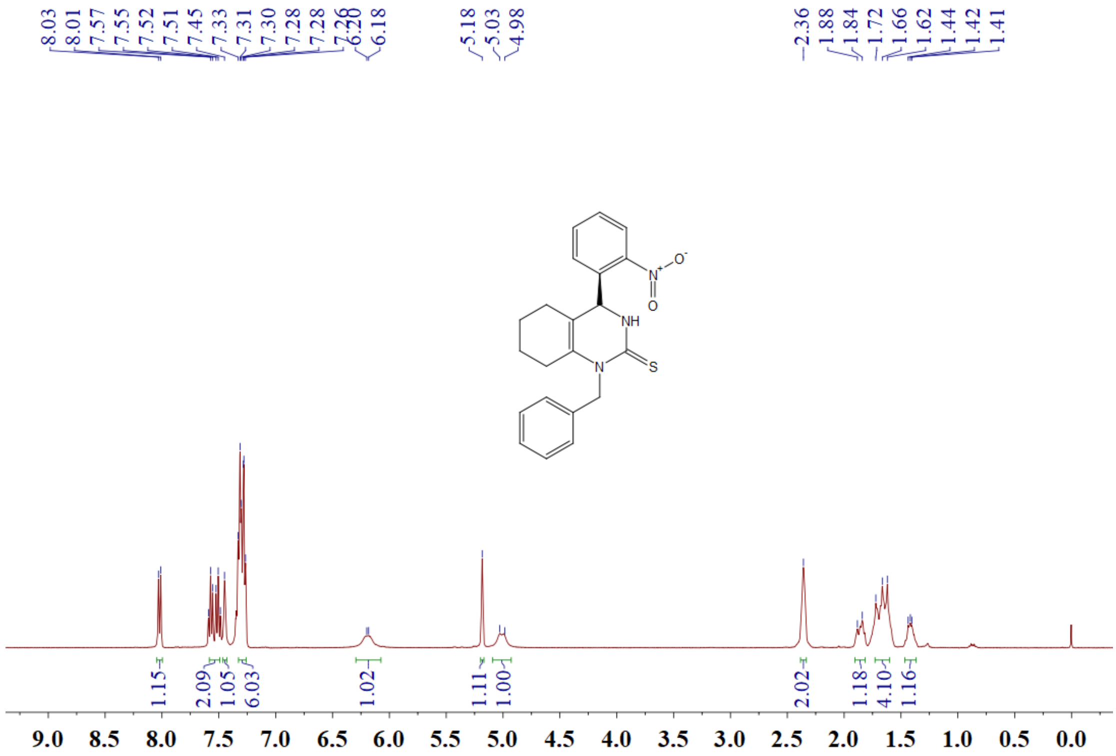
8.03
8.01
7.57
7.55
7.52
7.51
7.45
7.33
7.31
7.30
7.28
7.28
7.26
6.20
6.18
5.18
5.03
4.98
2.36
1.88
1.84
1.72
1.66
1.62
1.44
1.42
1.41
NH
N
S
N
O
O
1.15
2.09
1.05
6.03
1.02
1.11
1.00
2.02
1.18
4.10
1.16
9.0 8.5 8.0 7.5 7.0 6.5 6.0 5.5 5.0 4.5 4.0 3.5 3.0 2.5 2.0 1.5 1.0 0.5 0.0

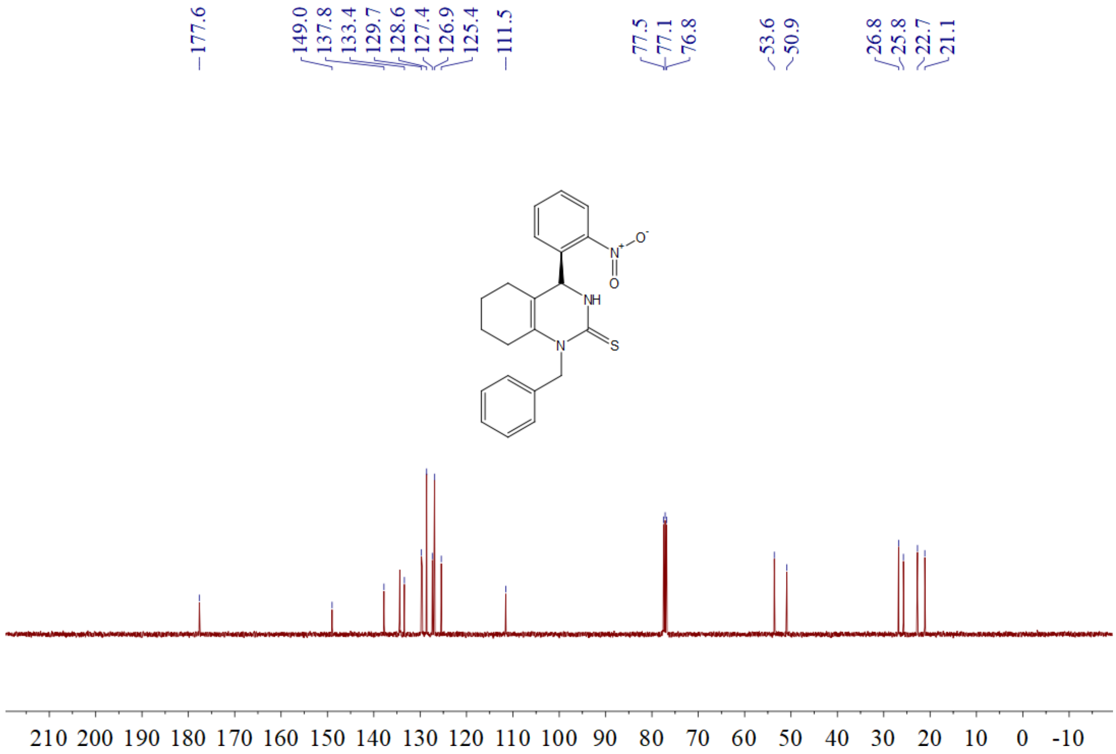
177.6
149.0
137.8
133.4
129.7
128.6
127.4
126.9
125.4
111.5
77.5
77.1
76.8
53.6
50.9
26.8
25.8
22.7
21.1
NH
N
S
N
O
O
210 200 190 180 170 160 150 140 130 120 110 100 90 80 70 60 50 40 30 20 10 0 -10

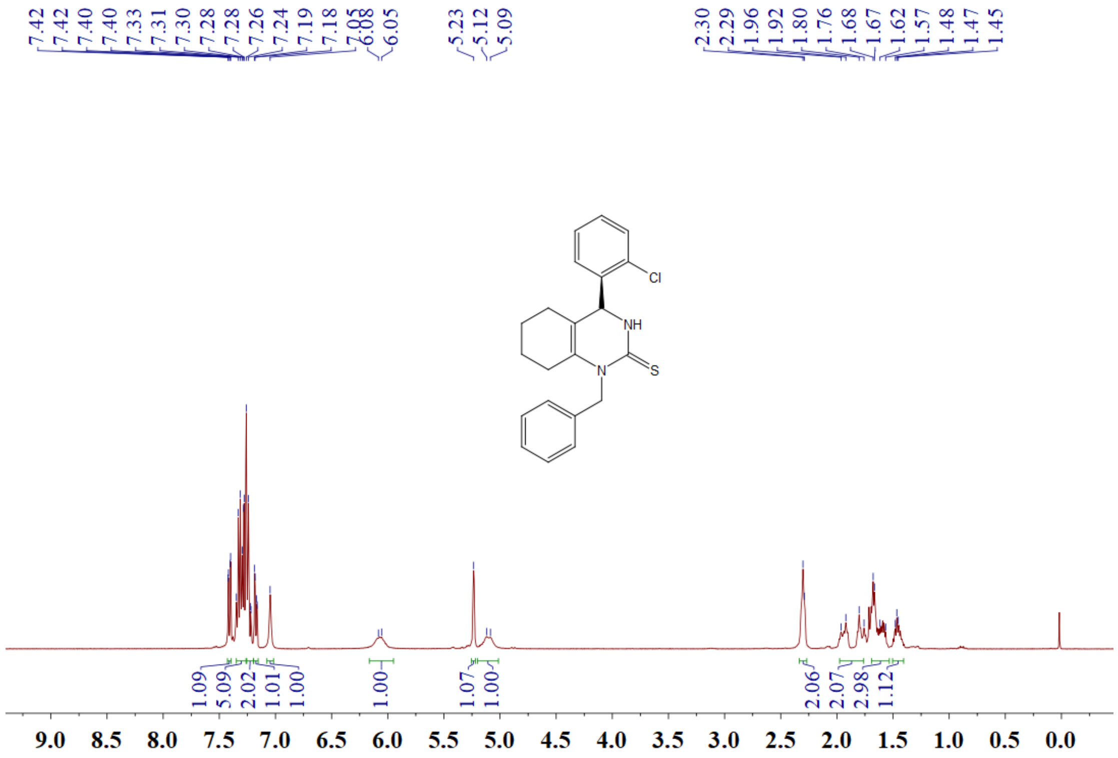
7.42
7.42
7.40
7.40
7.33
7.31
7.30
7.28
7.28
7.26
7.24
7.19
7.18
7.05
6.08
6.05
5.23
5.12
5.09
2.30
2.29
1.96
1.92
1.80
1.76
1.68
1.67
1.62
1.57
1.48
1.47
1.45
Cl
NH
N
S
1.09
5.09
2.02
1.01
1.00
1.00
1.07
1.00
2.06
2.07
2.98
1.12
9.0 8.5 8.0 7.5 7.0 6.5 6.0 5.5 5.0 4.5 4.0 3.5 3.0 2.5 2.0 1.5 1.0 0.5 0.0

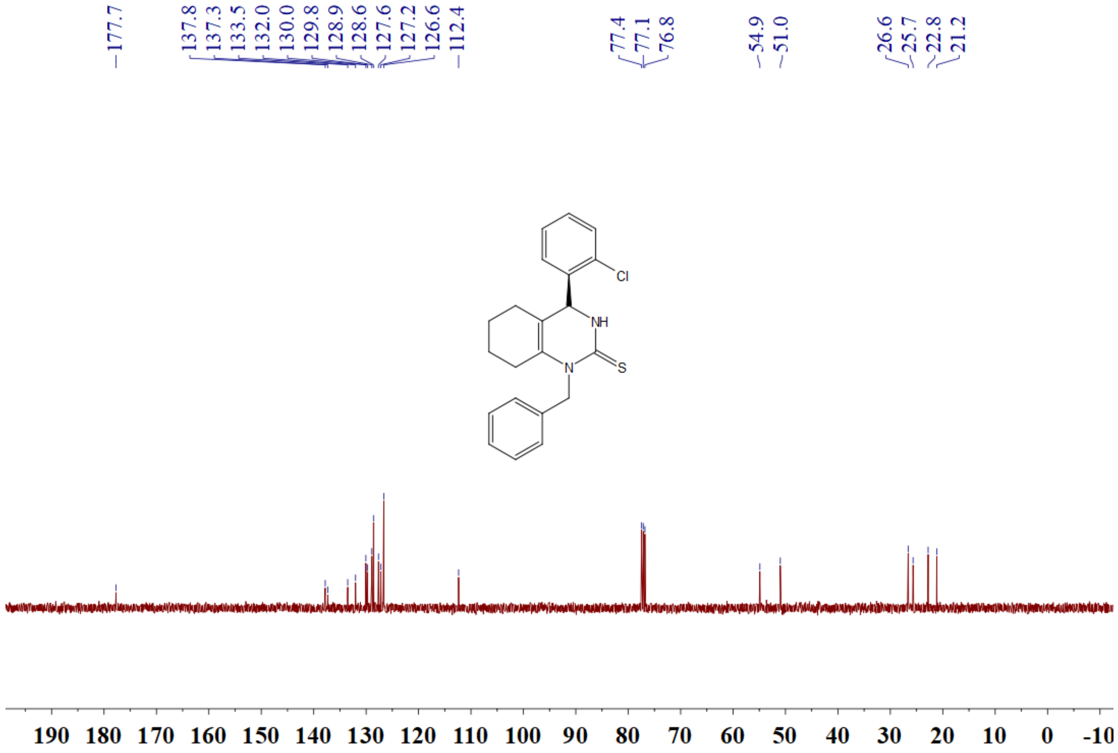
177.7
137.8
137.3
133.5
132.0
130.0
129.8
128.9
128.6
127.6
127.2
126.6
112.4
77.4
77.1
76.8
54.9
51.0
26.6
25.7
22.8
21.2
Cl
NH
N
S
190 180 170 160 150 140 130 120 110 100 90 80 70 60 50 40 30 20 10 0 -10

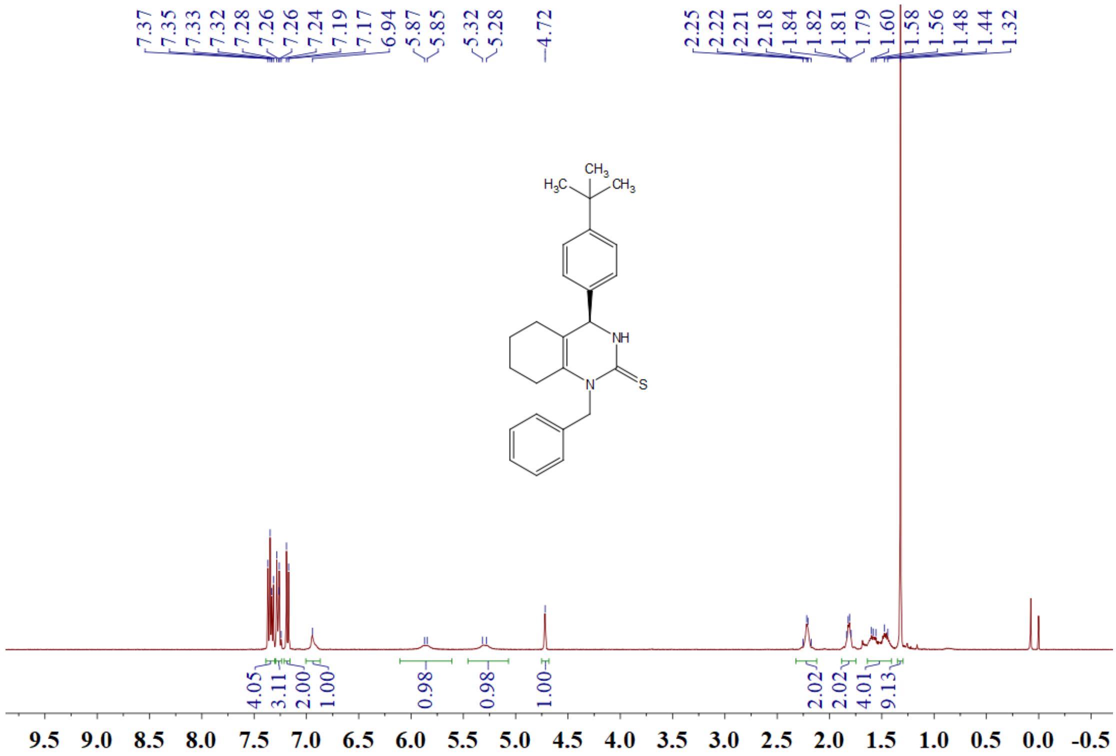
7.37 7.35 7.33 7.32 7.28 7.26 7.26 7.24 7.19 7.17 6.94 5.87 5.85 5.32 5.28 4.72
2.25 2.22 2.21 2.18 1.84 1.82 1.81 1.79 1.60 1.58 1.56 1.48 1.44 1.32
H_3C
CH_3
CH_3
NH
N
S
4.05 3.11 2.00 1.00 0.98 0.98 1.00 2.02 2.02 4.01 9.13
9.5 9.0 8.5 8.0 7.5 7.0 6.5 6.0 5.5 5.0 4.5 4.0 3.5 3.0 2.5 2.0 1.5 1.0 0.5 0.0 -0.5

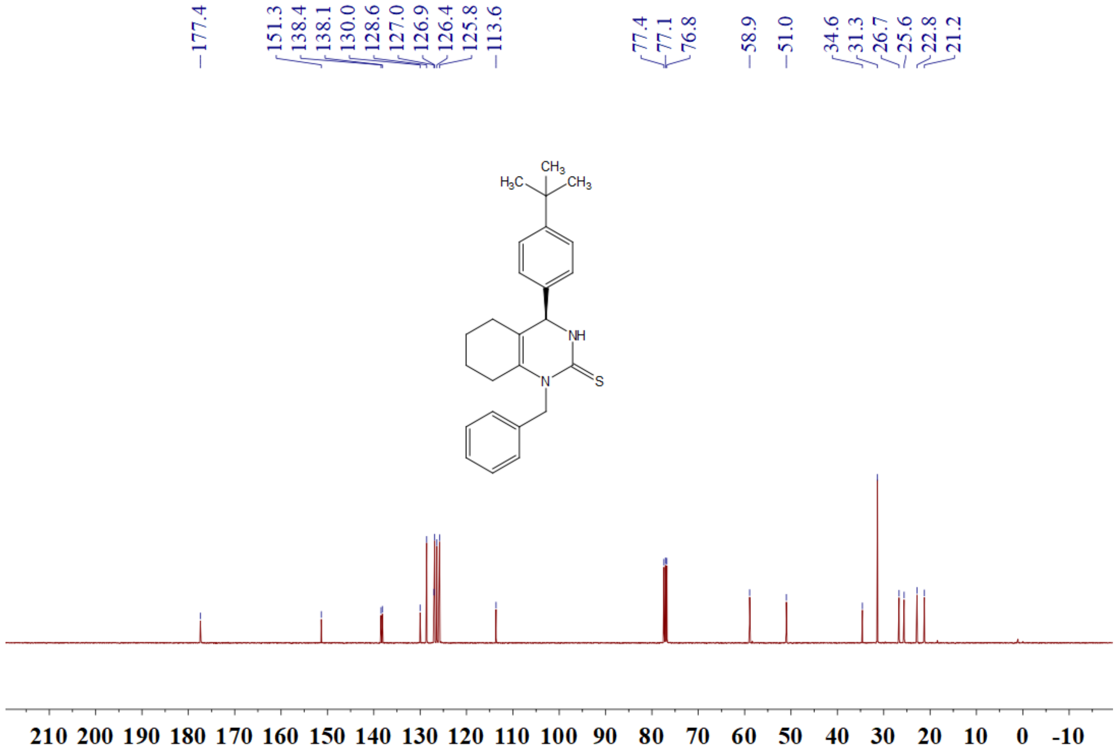
177.4
151.3 138.4 138.1 130.0 128.6 127.0 126.9 126.4 125.8 113.6
77.4 77.1 76.8
58.9
51.0
34.6 31.3 26.7 25.6 22.8 21.2
H_3C
CH_3
CH_3
NH
N
S
210 200 190 180 170 160 150 140 130 120 110 100 90 80 70 60 50 40 30 20 10 0 -10

III HPLC analysis

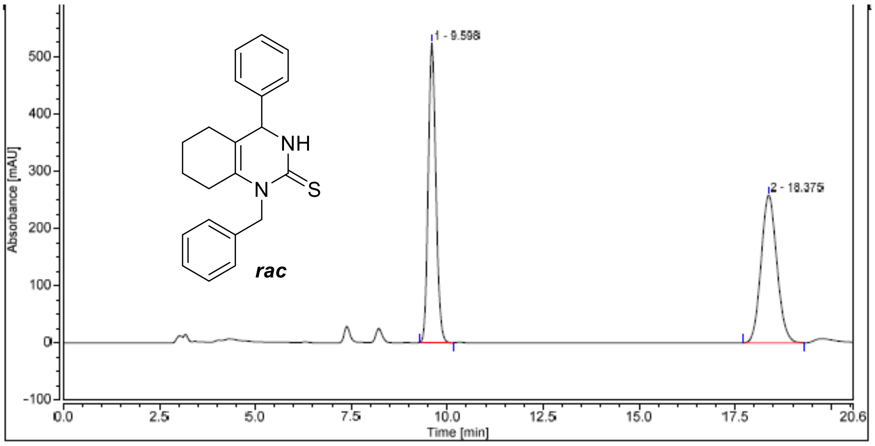

Integration Results							
No.	Peak Name	Retention Time min	Area mAU*min	Height mAU	Relative Area %	Relative Height %	Amount n.a.
1		9.598	121.033	523.676	49.63	66.97	n.a.
2		18.375	122.849	258.316	50.37	33.03	n.a.
Total:			243.881	781.992	100.00	100.00	

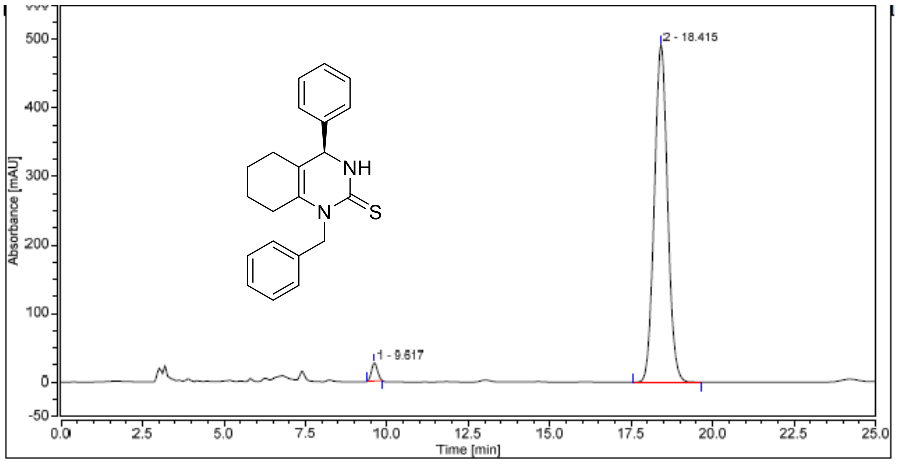

Integration Results							
No.	Peak Name	Retention Time min	Area mAU*min	Height mAU	Relative Area %	Relative Height %	Amount n.a.
1		9.617	5.584	26.324	2.35	5.08	n.a.
2		18.415	231.823	492.353	97.65	94.92	n.a.
Total:			237.407	518.676	100.00	100.00	

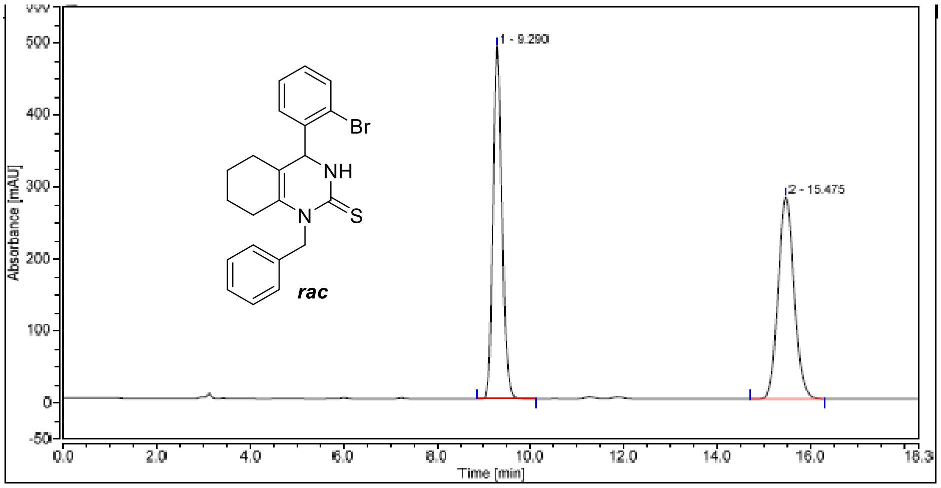

Integration Results							
No.	Peak Name	Retention Time min	Area mAU*min	Height mAU	Relative Area %	Relative Height %	Amount n.a.
1		9.290	110.609	488.672	49.94	63.54	n.a.
2		15.475	110.871	280.360	50.06	36.46	n.a.
Total:			221.480	769.032	100.00	100.00	

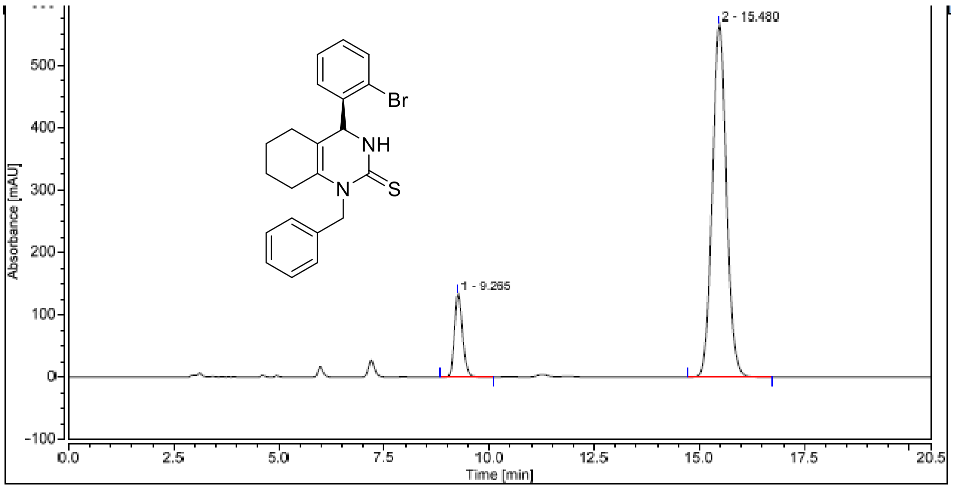

Integration Results							
No.	Peak Name	Retention Time min	Area mAU*min	Height mAU	Relative Area %	Relative Height %	Amount n.a.
1		9.265	30.185	133.170	11.90	19.07	n.a.
2		15.480	223.485	565.043	88.10	80.93	n.a.
Total:			253.669	698.212	100.00	100.00	

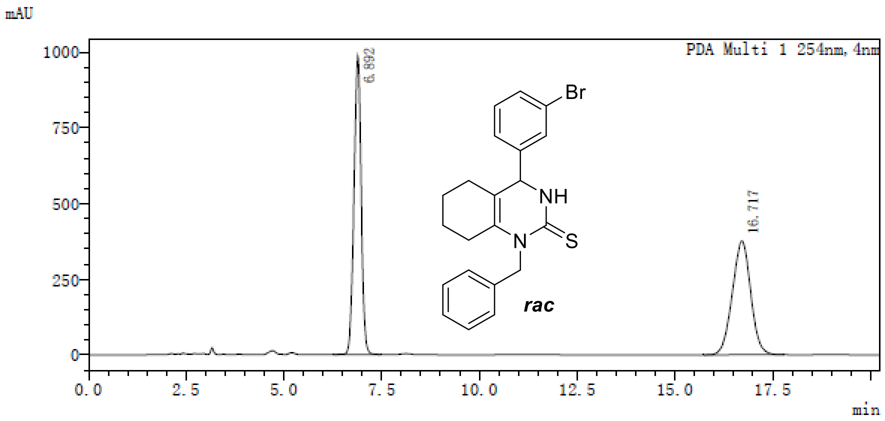

<峰表>

PDA Ch1 254nm

峰号	保留时间	面积	高度	面积%	高度%
1	6.892	12652770	985980	50.067	72.400
2	16.717	12618913	375878	49.933	27.600
总计		25271683	1361858	100.000	100.000

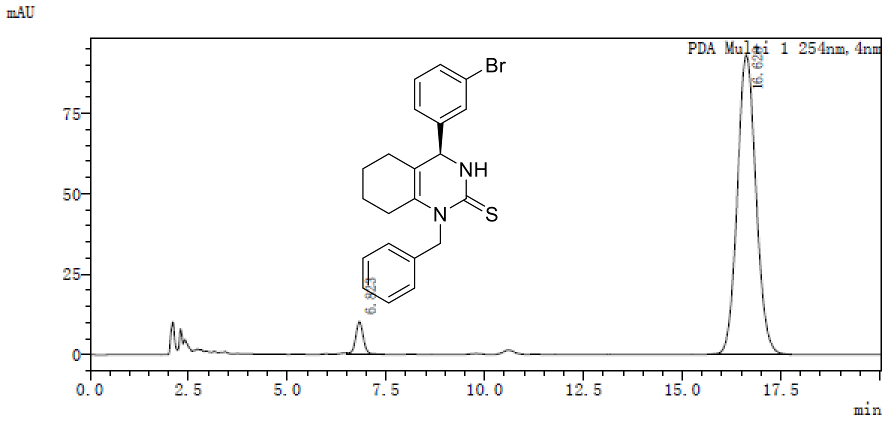

<峰表>

PDA Ch1 254nm

峰号	保留时间	面积	高度	面积%	高度%
1	6.823	137950	10155	4.268	9.852
2	16.628	3094121	92923	95.732	90.148
总计		3232072	103078	100.000	100.000

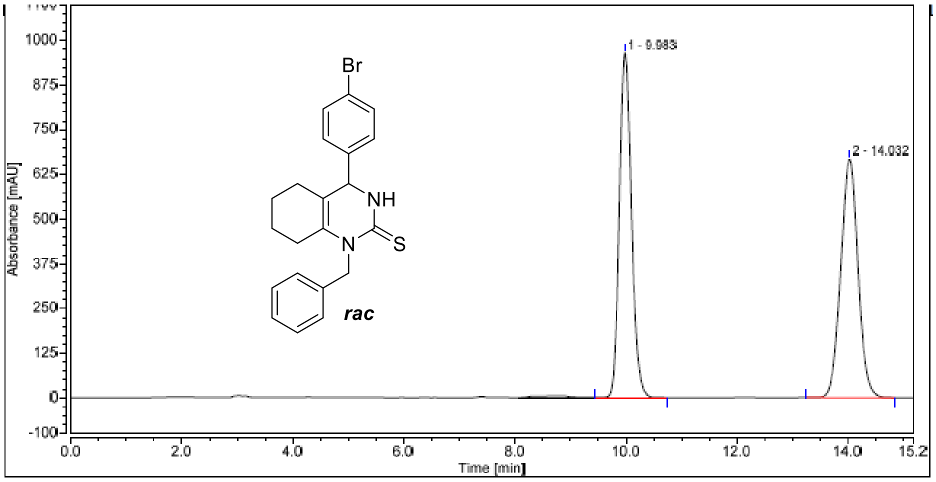

Integration Results							
No.	Peak Name	Retention Time min	Area mAU*min	Height mAU	Relative Area %	Relative Height %	Amount n.a.
1		9.983	242.184	964.838	50.07	59.06	n.a.
2		14.032	241.482	668.843	49.93	40.94	n.a.
**Total:**			**483.666**	**1633.681**	**100.00**	**100.00**	

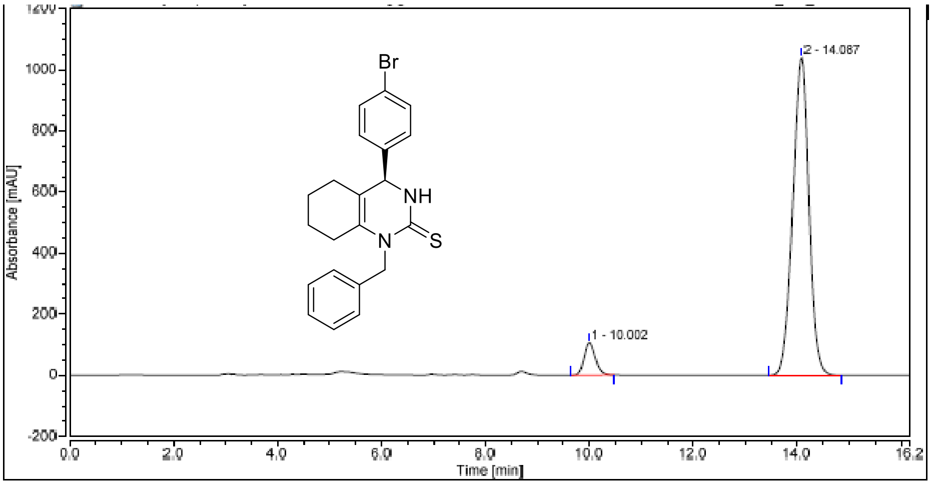

Integration Results							
No.	Peak Name	Retention Time min	Area mAU*min	Height mAU	Relative Area %	Relative Height %	Amount n.a.
1		10.002	26.509	106.201	6.58	9.27	n.a.
2		14.087	376.576	1039.589	93.42	90.73	n.a.
**Total:**			**403.085**	**1145.790**	**100.00**	**100.00**	

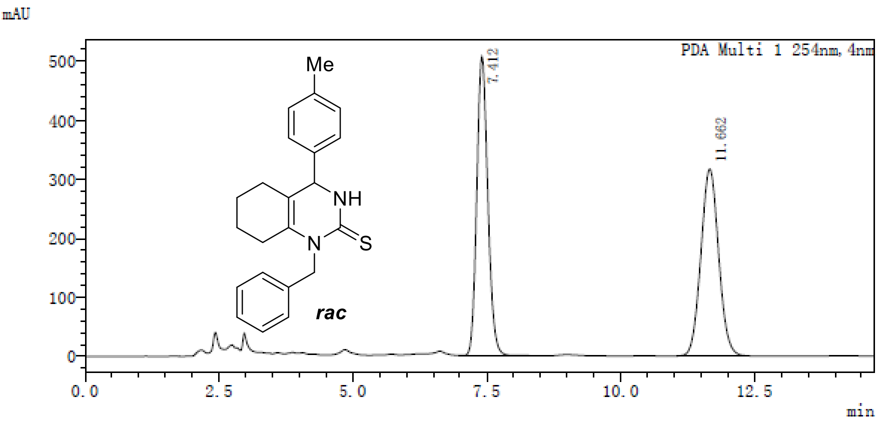

〈峰表〉

PDA Ch1 254nm

峰号	保留时间	面积	高度	面积%	高度%
1	7.412	7210578	506040	49.876	61.521
2	11.662	7246523	316505	50.124	38.479
总计		14457101	822546	100.000	100.000

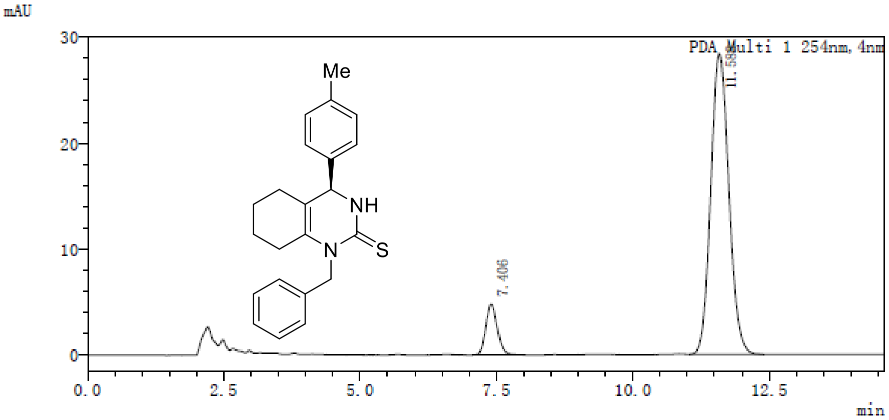

〈峰表〉

PDA Ch1 254nm

峰号	保留时间	面积	高度	面积%	高度%
1	7.406	68164	4771	9.593	14.395
2	11.588	642420	28370	90.407	85.605
总计		710584	33141	100.000	100.000

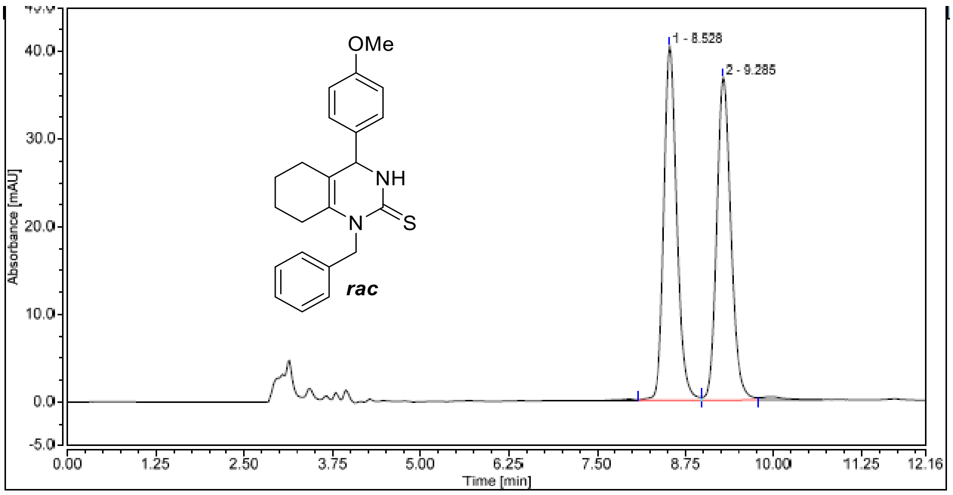

Integration Results							
No.	Peak Name	Retention Time min	Area mAU*min	Height mAU	Relative Area %	Relative Height %	Amount n.a.
1		8.528	8.544	40.406	50.13	52.31	n.a.
2		9.285	8.499	36.834	49.87	47.69	n.a.
**Total:**			**17.042**	**77.240**	**100.00**	**100.00**	

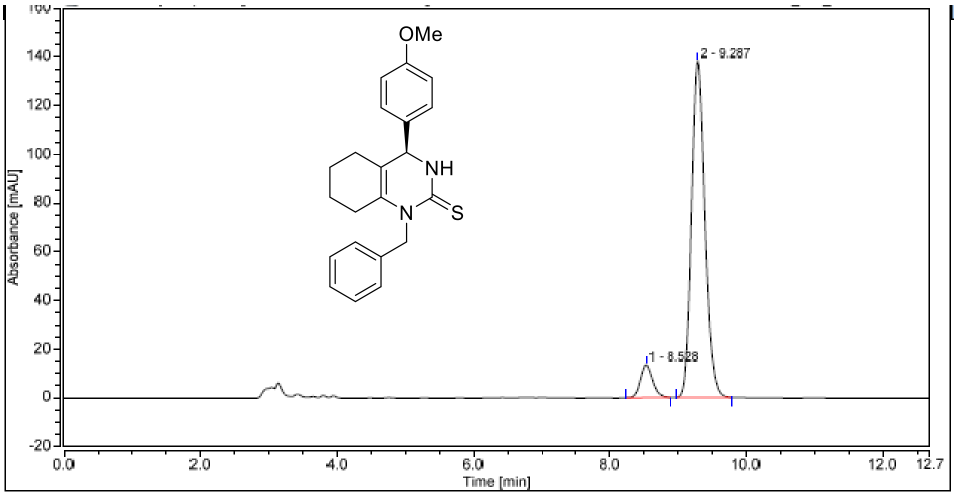

Integration Results							
No.	Peak Name	Retention Time min	Area mAU*min	Height mAU	Relative Area %	Relative Height %	Amount n.a.
1		8.528	2.800	13.500	8.21	8.90	n.a.
2		9.287	31.288	138.100	91.79	91.10	n.a.
**Total:**			**34.088**	**151.599**	**100.00**	**100.00**	

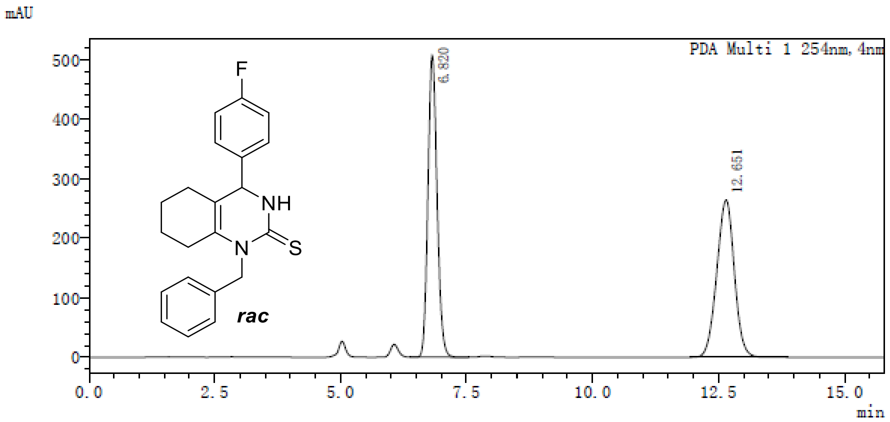

〈峰表〉
PDA Ch1 254nm

峰号	保留时间	面积	高度	面积%	高度%
1	6.820	6423215	506471	49.986	65.713
2	12.651	6426730	264264	50.014	34.287
总计		12849945	770735	100.000	100.000

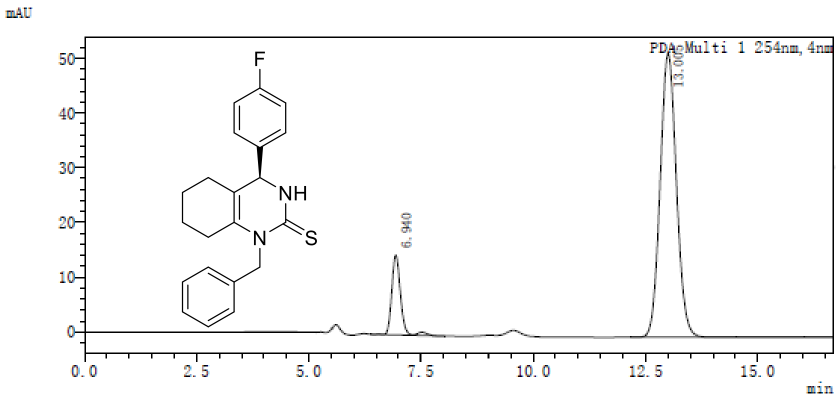

〈峰表〉
PDA Ch1 254nm

峰号	保留时间	面积	高度	面积%	高度%
1	6.940	203650	14641	13.434	22.031
2	13.005	1312267	51816	86.566	77.969
总计		1515917	66457	100.000	100.000

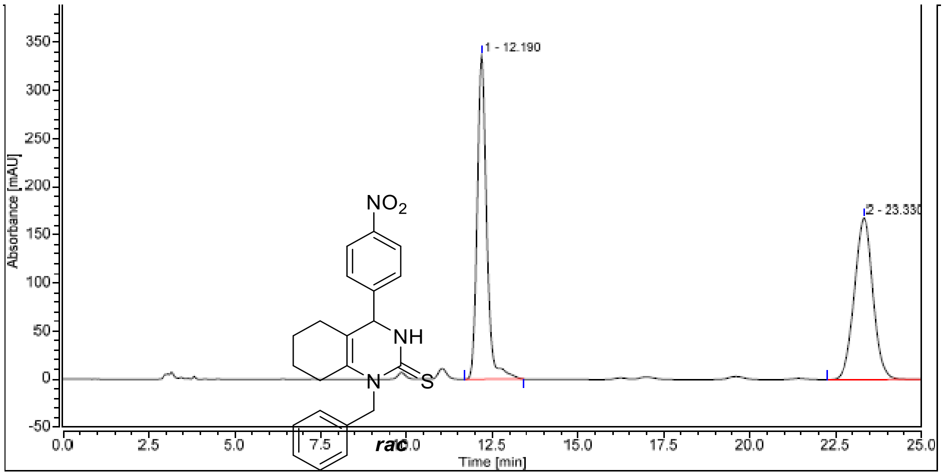

Integration Results							
No.	Peak Name	Retention Time min	Area mAU*min	Height mAU	Relative Area %	Relative Height %	Amount n.a.
1		12.190	110.006	337.164	50.99	66.71	n.a.
2		23.330	105.717	168.237	49.01	33.29	n.a.
Total:			215.723	505.400	100.00	100.00	

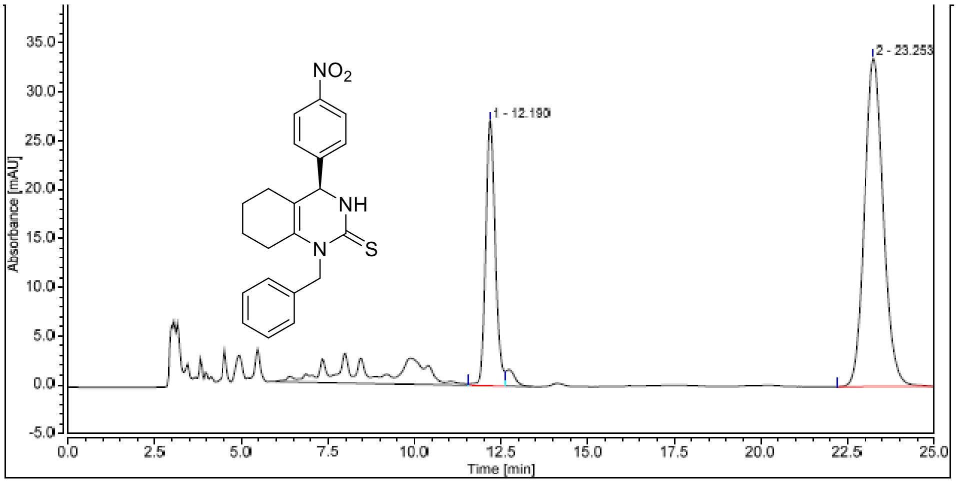

Integration Results							
No.	Peak Name	Retention Time min	Area mAU*min	Height mAU	Relative Area %	Relative Height %	Amount n.a.
1		12.190	8.622	27.114	28.83	44.67	n.a.
2		23.253	21.284	33.581	71.17	55.33	n.a.
Total:			29.907	60.695	100.00	100.00	

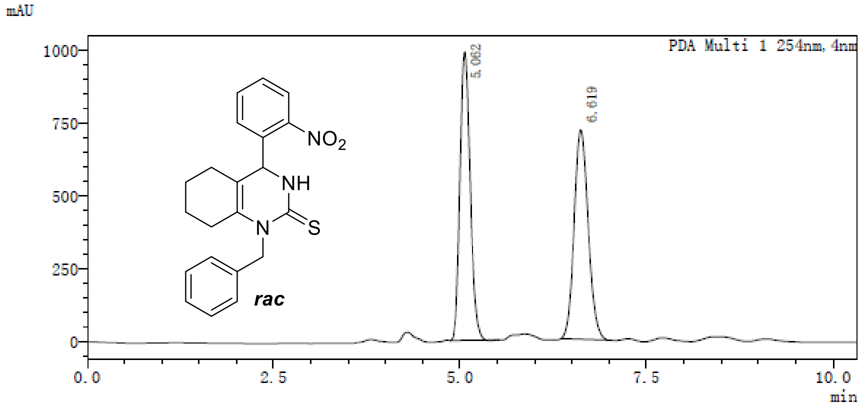

〈峰表〉
PDA Ch1 254nm

峰号	保留时间	面积	高度	面积%	高度%
1	5.062	9311879	986257	49.855	57.989
2	6.619	9365977	714519	50.145	42.011
总计		18677855	1700776	100.000	100.000

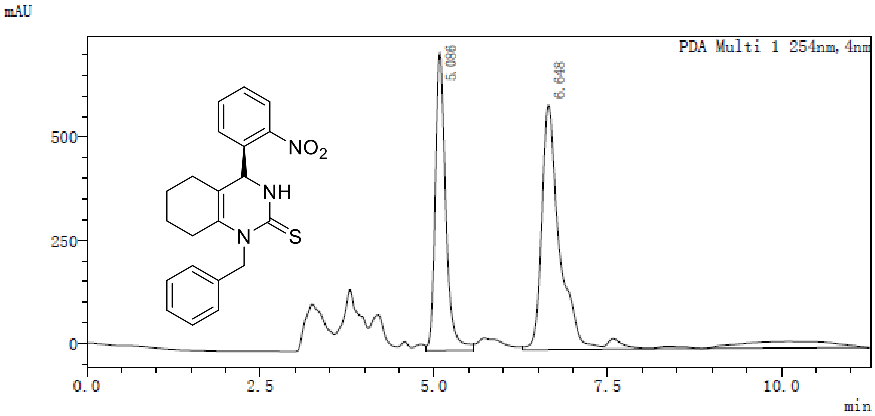

〈峰表〉
PDA Ch1 254nm

峰号	保留时间	面积	高度	面积%	高度%
1	5.086	7733980	720440	38.215	54.880
2	6.648	12503996	592312	61.785	45.120
总计		20237976	1312752	100.000	100.000

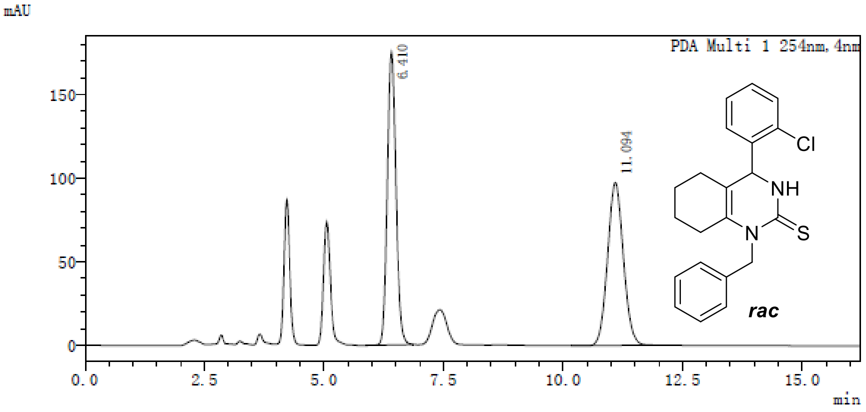

<峰表>

PDA Ch1 254nm

峰号	保留时间	面积	高度	面积%	高度%
1	6.410	2188387	174931	49.328	64.172
2	11.094	2248010	97667	50.672	35.828
总计		4436397	272599	100.000	100.000

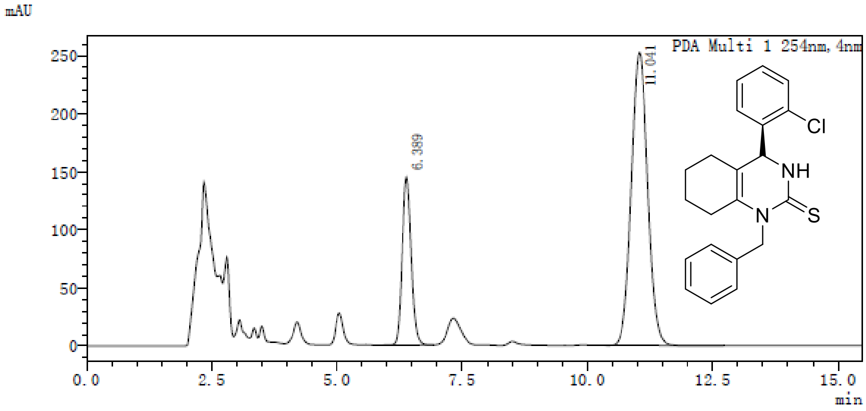

<峰表>

PDA Ch1 254nm

峰号	保留时间	面积	高度	面积%	高度%
1	6.389	1815045	144681	23.971	36.427
2	11.041	5756637	252501	76.029	63.573
总计		7571682	397182	100.000	100.000

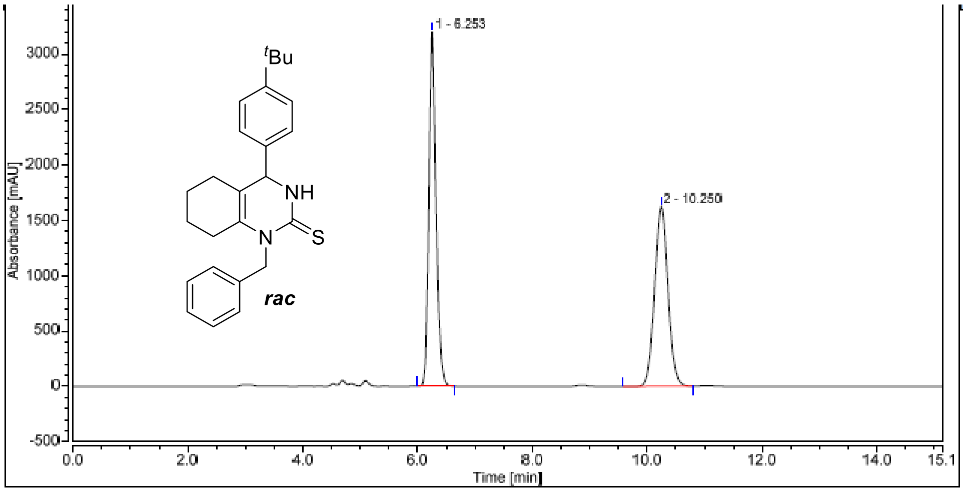

Integration Results							
No.	Peak Name	Retention Time<br>min	Area<br>mAU*min	Height<br>mAU	Relative Area<br>%	Relative Height<br>%	Amount<br>n.a.
1		6.253	455.216	3200.057	50.97	66.32	n.a.
2		10.250	437.818	1624.997	49.03	33.68	n.a.

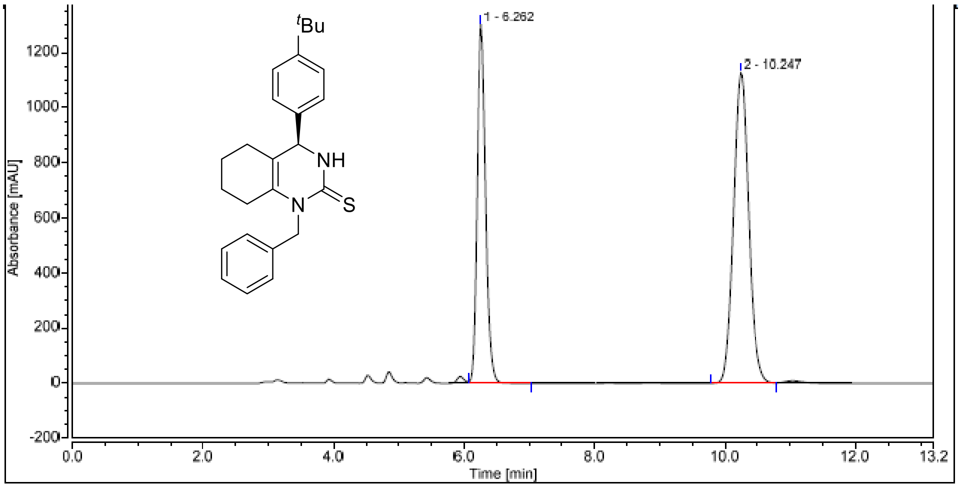

Integration Results							
No.	Peak Name	Retention Time<br>min	Area<br>mAU*min	Height<br>mAU	Relative Area<br>%	Relative Height<br>%	Amount<br>n.a.
1		6.262	199.200	1300.164	39.52	53.55	n.a.
2		10.247	304.877	1127.859	60.48	46.45	n.a.
**Total:**			**504.077**	**2428.023**	**100.00**	**100.00**	
